# Deep Learning‐Driven Exploration of Pyrroloquinoline Quinone Neuroprotective Activity in Alzheimer's Disease

**DOI:** 10.1002/advs.202308970

**Published:** 2024-03-07

**Authors:** Xinuo Li, Yuan Sun, Zheng Zhou, Jinran Li, Sai Liu, Long Chen, Yiting Shi, Min Wang, Zheying Zhu, Guangji Wang, Qiulun Lu

**Affiliations:** ^1^ Jiangsu Provincial Key Laboratory of Drug Metabolism and Pharmacokinetics State Key Laboratory of Natural Medicines China Pharmaceutical University Nanjing 211166 China; ^2^ Department of Computer Science RWTH Aachen University 52074 Aachen Germany; ^3^ Affiliated Brain Hospital of Nanjing Medical University Nanjing 210029 China; ^4^ School of Pharmacy The University of Nottingham Nottingham NG7 2RD UK

**Keywords:** Alzheimer's disease, deep learning, neuroprotective activities, pyrroloquinoline quinones

## Abstract

Alzheimer's disease (AD) is a pressing concern in neurodegenerative research. To address the challenges in AD drug development, especially those targeting Aβ, this study uses deep learning and a pharmacological approach to elucidate the potential of pyrroloquinoline quinone (PQQ) as a neuroprotective agent for AD. Using deep learning for a comprehensive molecular dataset, blood–brain barrier (BBB) permeability is predicted and the anti‐inflammatory and antioxidative properties of compounds are evaluated. PQQ, identified in the Mediterranean‐DASH intervention for a diet that delays neurodegeneration, shows notable BBB permeability and low toxicity. In vivo tests conducted on an Aβ₁₋₄₂‐induced AD mouse model verify the effectiveness of PQQ in reducing cognitive deficits. PQQ modulates genes vital for synapse and anti‐neuronal death, reduces reactive oxygen species production, and influences the SIRT1 and CREB pathways, suggesting key molecular mechanisms underlying its neuroprotective effects. This study can serve as a basis for future studies on integrating deep learning with pharmacological research and drug discovery.

## Graphical abstract

The graphics highlight the role of deep learning in identifying PQQ as a potential agent for AD treatment, through the inhibition of inflammation and reduction of oxidation, to exhibit neuroprotection. Clinical data show lower PQQ levels in AD patients, suggesting consumption of PQQ‐rich foods as an important nutrition strategy for those at risk of developing AD at any stage of life.

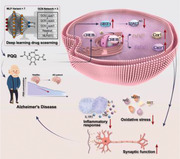

## 1 Introduction

Alzheimer's disease (AD), the leading form of dementia, is a complex neurodegenerative disorder closely associated with aging. The neuropathological signatures of AD are characterized by the presence of extracellular deposits of amyloid β (Aβ) and intracellular aggregates of hyperphosphorylated Tau proteins.^[^ ^1^ ^]^ Currently, drugs targeting Aβ have two main limitations: their limited efficacy, which does not sufficiently alleviate the clinical symptoms of patients with AD, and their significant toxicity and side effects, making them unsuitable for extended use.^[^ ^2^ ^]^ Thus, there is an urgent need to find a potent medication with low toxicity for daily administration to AD patients.

Despite the focus on Aβ‐targeted therapies, current drugs including donepezil and lecanemab have not consistently shown effectiveness in alleviating AD symptoms in clinical trials.^[^ ^3^ ^]^ Moreover, some of these drugs, particularly lecanemab—an anti‐Aβ monoclonal antibody therapeutic—raise concerns because of their pronounced toxicities and limited permeability through the blood–brain barrier (BBB).^[^ ^4^ ^]^ Given the long‐term therapeutic needs of AD patients, the quest for an orally administered, highly effective, and low‐toxicity anti‐AD agent that targets alternative molecular pathways has led to the discovery of novel, potent, and well‐tolerated anti‐AD compounds.

Recent research indicates that neuroinflammation and oxidative stress play key roles in the progression of AD.^[^ ^5^ ^]^ Neuroinflammation in the central nervous system, primarily driven by microglial and astrocytic responses, increases the levels of proinflammatory cytokines, contributing to cognitive decline.^[^ ^6^ ^]^ There is a correlation between elevated cerebrospinal fluid cytokine levels and AD progression.^[^ ^7^ ^]^ This inflammation also increases reactive oxygen species (ROS) production, resulting in enhanced oxidative stress, which is apparent in the brains of patients with AD,^[^ ^8^ ^]^ and affects Aβ accumulation and Tau hyperphosphorylation.^[^ ^9^, ^10^ ^]^ Therefore, addressing neuroinflammation and oxidative stress is essential in the development of anti‐AD therapeutics.

Many modern anti‐AD drugs, including memantine and donepezil, exhibit significant toxicities. Epidemiological studies underscore the protective ability of compounds, particularly those sourced from plants and diets, against AD.^[^ ^11^, ^12^ ^]^ By scavenging ROS, modulating cytokines, and strengthening neuronal antioxidative defenses, these agents offer promising therapeutic and preventive strategies for AD with minimal toxicity.^[^ ^13^ ^]^

In recent years, the integration of deep learning computational models into the pharmaceutical domain has emerged as a significant advancement, which has led to the prediction of compound properties and activities,^[^ ^14^ ^]^ forecasting of target interactions,^[^ ^15^ ^]^ drug screening,^[^ ^16^ ^]^ and innovative design of new drugs.^[^ ^17^ ^]^ These models offer considerable promise, especially in navigating the complexities of biomedical data. Neural networks, which are foundational to these models, not only expedite the processing of high‐dimensional chemical and biological data but also reveal previously hidden patterns.^[^ ^18^ ^]^ This facilitates ground‐breaking discoveries in drug development and related fields. By training these models on vast databases, we can predict and create target molecules. This is instrumental in identifying compounds that exhibit specific therapeutically relevant properties.^[^ ^19^, ^20^ ^]^

The MIND diet, which integrates the best elements from the Mediterranean and DASH diets, is designed to reduce dementia risk. It prioritizes plants, nuts, berries, fish, and olive oil, all known for their potential cognitive benefits.^[^ ^21^, ^22^, ^23^ ^]^ Research increasingly suggests that specific diets and nutrients may delay Alzheimer's‐related cognitive decline.^[^ ^24^, ^25^ ^]^ However, the complex mechanisms of the brain that are affected remain unclear. The MIND diet, centered around cognitive health, opens valuable avenues for understanding the complex mechanisms underlying AD pathogenesis and establishes a basis for dietary regimens designed to alleviate the effects of AD.

Pyrroloquinoline quinone (PQQ), recognized as an oxidoreductase coenzyme in mammals, has broad therapeutic potential, from anti‐inflammatory to neuroprotective effects.^[^ ^26^, ^27^, ^28^ ^]^ Recent research has highlighted the neuroprotective qualities of PQQ, including its capacity to cross the BBB,^[^ ^29^ ^]^ provide protection in stroke models,^[^ ^30^ ^]^ and reduce Aβ‐induced toxicity in SH‐SY5Y cells.^[^ ^31^ ^]^ PQQ has shown protective effects against various neuronal injuries, such as glutamate‐induced damage and 6‐hydroxydopamine‐induced neurotoxicity.^[^ ^32^, ^33^ ^]^ However, the exact role and molecular mechanisms of PQQ in the treatment of AD remain elusive.

In this study, we strategically leveraged the strengths of a deep learning pipeline with pharmacological principles to identify PQQ, a potent and low‐toxicity compound sourced from food, suitable for oral consumption. Using a comprehensive dataset of molecular structures, our deep learning models were trained to predict BBB permeability as well as anti‐inflammatory and antioxidative properties. Our research might underscore the potential of deep learning in harnessing existing data to identify promising therapeutic compounds, thereby laying foundational methods for innovative drug discovery avenues. Moving forward, PQQ could be considered a vitamin‐like supplement for AD patients or as a prophylactic for those at risk. Additionally, our findings might bridge the gap between daily dietary practices and the therapeutic implications for AD.

## 2 Results

### 2.1 Deep Learning‐Based Drug Screening for AD

To discover novel anti‐inflammatory and antioxidant drugs for AD, we developed a series of deep‐learning models. Each model is designed to predict specific attributes, such as a compound's ability to traverse the BBB, its anti‐inflammatory potential, and its antioxidative capacity. We leveraged an extensive dataset featuring Simplified Molecular Input Line Entry System representations, tagging each molecule with binary labels spanning 10 unique attributes, such as BBB permeability, and both anti‐inflammatory and antioxidative properties (Figure S1a, Supporting Information). These binary labels, denoted by 0 or 1, indicate the lack or presence of a specific characteristic, respectively. For ranking purposes, we determined the cumulative score of each molecule by summing its attribute values. Compounds scoring 5≥ were considered to possess a notable number of desired attributes, positioning them as prime contenders for further assessments.

**Figure 1:**
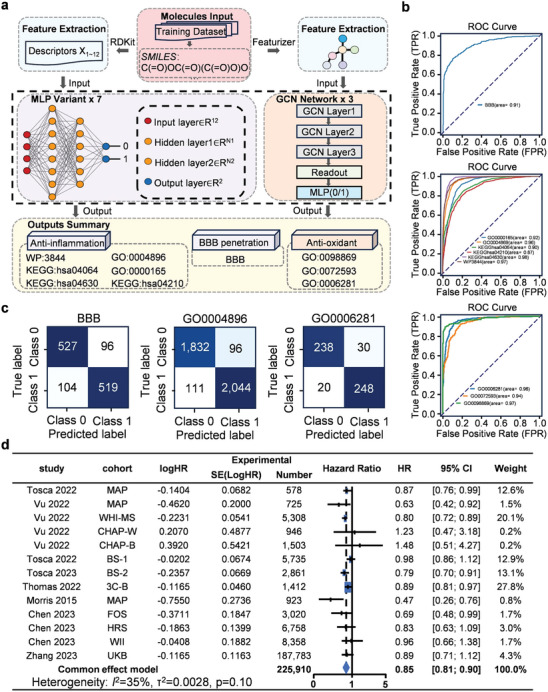
Drug‐screening model of deep learning for Alzheimer's disease (AD) and protective effect of the Mediterranean dietary pattern lowers the risk of AD. a) Dual‐path modeling architecture. Descriptors X_1‐12_, extracted via RDKit, feed our Multi‐Layer Perceptron variants designed for BBB and anti‐inflammatory properties. Conversely, molecular graphs crafted using deepchem's MolGraphConvFeaturizer act as inputs for the Graph Convolutional Network models designed for the antioxidant properties. b) Receiver operating characteristic curves demonstrating the predictive performance of our models. c) Confusion matrices for BBB and the anti‐inflammatory property based on the test set results and the antioxidative property obtained using 5‐fold cross‐validation. For each channel, the matrix represents the validation results of the fold with the highest F1 score. d) Meta‐analysis: A meta‐analysis is presented, showcasing the protective effect of the Mediterranean‐DASH Diet Intervention for Neurodegenerative Delay (MIND diet) against AD across diverse cohorts.

Our computational aim was to pinpoint potential compounds for AD treatment. Our dual‐path modeling approach integrates molecular descriptors from RDKit (https://www.rdkit.org/) into our set of Multi‐Layer Perceptron (MLP)^[^ ^34^ ^]^ models, distinctly crafted to predict BBB and anti‐inflammatory properties (**Figure** **1**a). Conversely, molecular graphs created using DeepChem's MolGraphConvFeaturizer^[^ ^35^ ^]^ serve as the input for the Graph Convolutional Networks (GCN)^[^ ^36^ ^]^ models, optimized for antioxidative property predictions. The receiver operating characteristic (ROC) curves emphasize the effectiveness of our modeling choices. Specifically, MLP models predict BBB permeability and six anti‐inflammatory attributes, while the GCN models can identify three antioxidative properties (Figure 1b). Moreover, in‐depth performance metrics validated the competence of our models, with confusion matrices offering insight into their classification efficacy (Figure 1c; Figure S1b–h, Supporting Information).

### 2.2 Mediterranean Dietary Pattern Reduces AD Risk

Recent studies suggest that adherence to the MIND diet may be associated with enhanced cognitive function in older adults.^[^ ^37^, ^38^ ^]^ To further validate the neuroprotective effect of MIND, we conducted a meta‐analysis using data reported globally from multiple races (Figure S2a, Supporting Information). The results showed that the MIND diet may protect against AD (hazard ratio 0.85, 95% CI 0.81–0.90, I^2^ 35.1%), indicating the potential of the MIND dietary pattern to offer protection against AD (Figure 1d).

### 2.3 Identification of PQQ as an Anti‐AD Agent from Dietary Patterns

**Figure 2:**
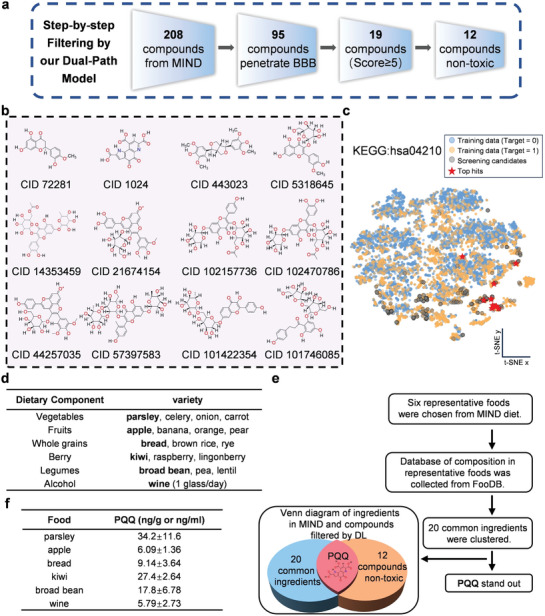
Deep learning‐guided identification of pyrroloquinoline quinone (PQQ) from dietary patterns as a potential therapeutic candidate for Alzheimer's disease (AD). a) MIND‐Derived Compound Screening Workflow: A schematic of the comprehensive screening process for compounds sourced from the Mediterranean‐DASH Diet Intervention for Neurodegenerative Delay (MIND diet) using our dual‐path model. b) Structures of non‐toxic compounds: Provides visual representations of the structures of the 12 non‐toxic compounds that successfully passed through the model's filtration process. c) Two‐dimensional t‐SNE visualization of the original dataset compounds employed in this work for the target Kyoto Encyclopedia of Genes and Genomes: has 0 4210, excluding any samples generated via augmentation techniques such as SMOTE and ADASYN. T‐SNE plots were generated with perplexity 30 and a maximal number of iterations 1000. The 12 highly scored non‐toxic compounds ultimately selected are marked and showcased in the plot as “top hits.” d) The food categories recommended by the MIND diet. e) Workflow for screening food components with other characters. f) Content of PQQ in Representative Foods: Provides information on the content of PQQ in six representative foods, shedding light on its presence in various dietary sources.

To understand how the MIND diet mitigates AD risk, our primary objective was to identify the essential components of this dietary pattern that contribute to reduced AD risk. We cumulated a total of 208 compounds based on the MIND diet recommendations (Table S3, Supporting Information). These compounds were evaluated using our deep learning‐based screening framework (**Figure** **2**a). Initially, we assessed BBB permeability and found that 113 of the 208 compounds could not cross the BBB, leaving 95 compounds with substantial BBB permeability. Among these, 19 compounds displayed robust anti‐inflammatory and antioxidative activities (score ≥ 5) (Table S4, Supporting Information). A subsequent search in PubChem refined our selection to 12 low‐toxicity compounds for a detailed analysis (Figure 2b). To validate the robustness of our methodology, we used t‐SNE visualization. The visualizations depicted a notable overlap between our training dataset and the 208 MIND diet‐derived screening candidates, emphasizing that our screening remained within the model's confidence domain (Figure 2c; Figure S3a–i, Supporting Information). Notably, our model identified naringenin^[^ ^39^, ^40^ ^]^ and hesperetin^[^ ^41^, ^42^ ^]^ for their potent anti‐inflammatory and antioxidant capabilities. In contrast, specific compounds, namely, CID:102 470 786, CID:102 157 736, and CID:101 746 085, lacked such properties. This alignment with existing literature accentuates the accuracy and reliability of our modeling framework.

To further determine the key compounds in the MIND diet that curtail AD onset and progression, we categorized dietary components based on MIND diet guidelines and showed representative food items for each group. Using the Food Database FooDB (https://foodb.ca/), we conducted an exhaustive ingredient analysis for each representative item, identifying 20 unique ingredients from the six presented items (Figure S2b, Supporting Information). After cross‐referencing these 20 unique ingredients with the 12 non‐toxic compounds identified through deep learning, PQQ emerged (Figure 2e,f). This indicates that PQQ might be a key element in the MIND dietary regimen for mitigating AD risk.

### 2.4 Reduction of Plasma PQQ Levels in AD Patients

**Figure 3:**
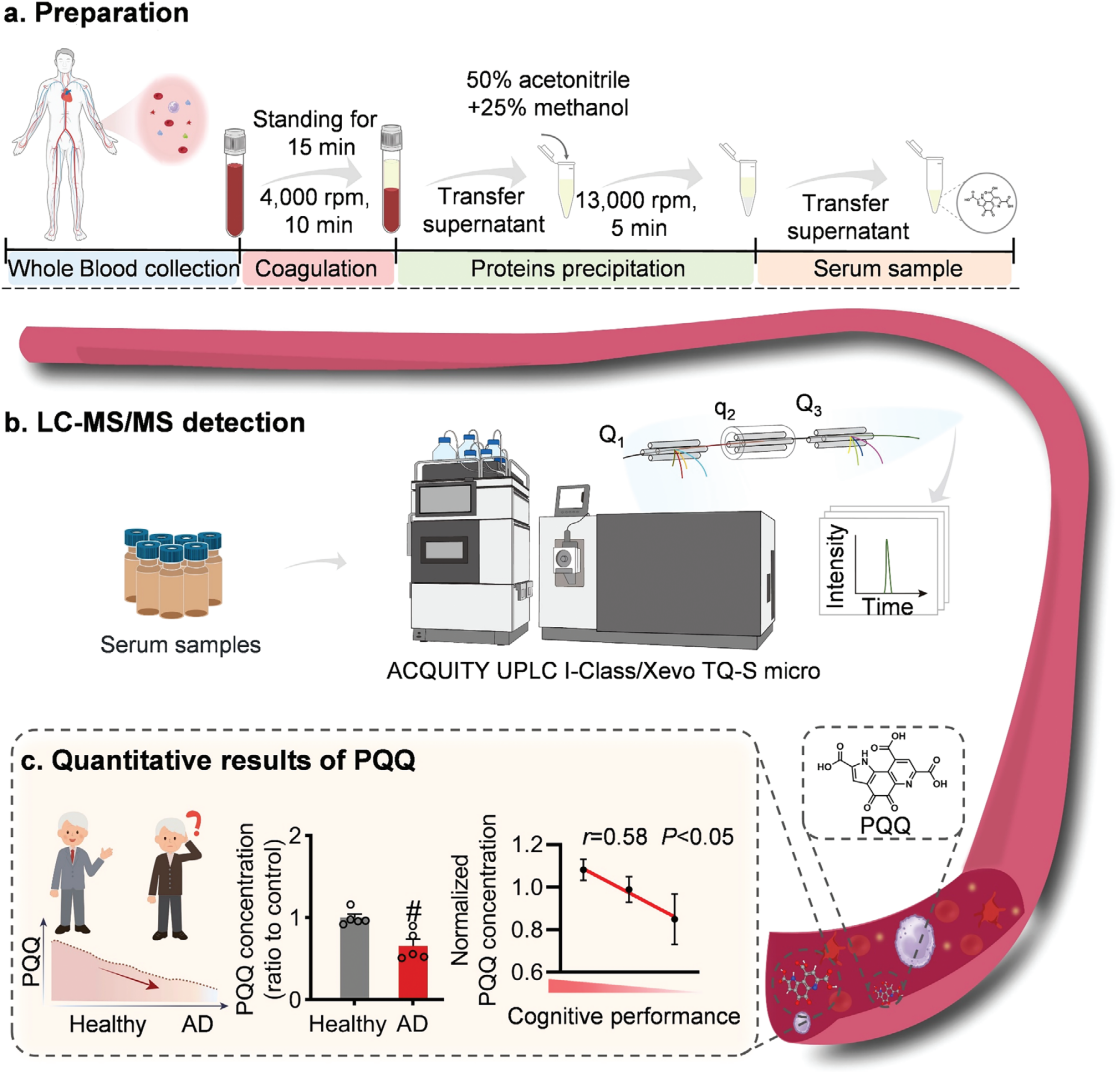
Declined levels of plasma pyrroloquinoline quinone (PQQ) in Alzheimer's disease (AD) patients. a) Schematic detailing the standard procedure for sample collection. b) Flowchart illustrating the steps involved in liquid chromatography‐tandem mass spectrometry analysis. c) Comparative PQQ concentrations in plasma samples obtained from healthy donors versus AD patients. Each dot is an individual donor (*n* = 5). ^#^ *p* < 0.05 versus healthy controls.

To further establish the correlation between PQQ levels and AD severity, we measured PQQ levels in plasma samples. Upon recruiting patients with AD, we collected their plasma (**Figure** **3**a). Given the inherent challenges in detecting trace amounts of PQQ in plasma, we adopted a standard measurement technique using liquid chromatography‐tandem mass spectrometry (LC‐MS/MS) (Figure 3b). Remarkably, using LC‐MS/MS‐based relative quantification—as indicated by the peak areas of the corresponding ion pairs—we found that the PQQ concentration in the plasma of patients with AD is significantly lower than that in healthy individuals (Figure 3c). Subsequent analysis indicated that the concentration of PQQ was negatively correlated with cognitive performance measures in patients with AD. This suggests that there is a tendency for PQQ levels to decrease as the severity of AD increases. This result quantitatively supports the observed decrease in PQQ levels among patients with AD.

### 2.5 PQQ Alleviates Cognitive Dysfunction In Vivo

**Figure 4:**
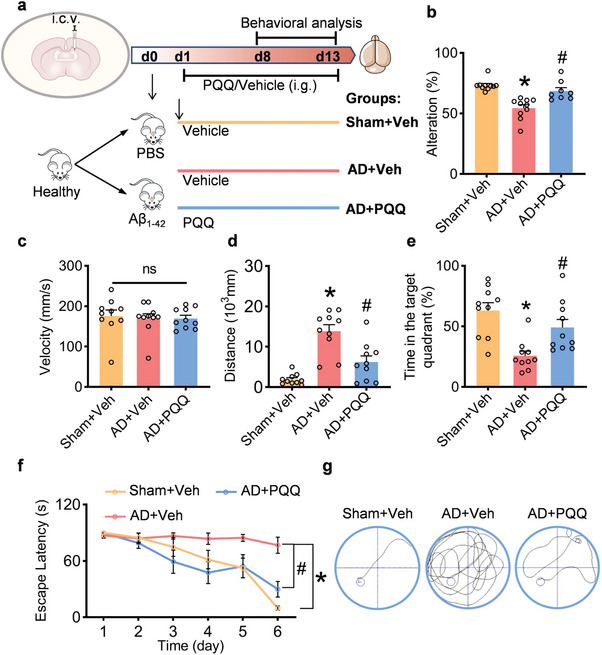
Pyrroloquinoline quinone (PQQ) alleviates cognitive dysfunction in vivo. a) Schematic of the experimental design (*n* = 10). b) Evaluation of spontaneous alternation in the Y‐maze for control mice compared with Alzheimer's disease (AD) mice after 7‐day treatment with either vehicle or PQQ. c) The average swimming speed among all groups. d) The distance to reach the platform in the probe. e) The percentage of time spent in the target quadrant in the probe. f) The latency to locate the platform during the experiment. g) Representative path tracings in the probe trial. Data are shown as mean ± SEM. ^*^ *p* < 0.05 versus control group; ^#^ *p* < 0.05 versus AD group.

To assess the therapeutic efficacy of PQQ in ameliorating cognitive dysfunction associated with AD, PQQ was administered orally to an AD mouse model induced by Aβ₁₋₄₂ for a consecutive 14‐day period (**Figure** **4**a). Short‐term memory was evaluated using a Y‐maze analysis. This test revealed that PQQ restored spontaneous alternation in Aβ₁₋₄₂‐induced AD mice, suggesting a remarkable improvement in the short‐term memory deficits observed in AD mice (Figure 4b). Spatial learning and memory were further examined using the Morris water maze analysis. No significant difference in swimming velocity was observed among the three mouse groups, confirming the reliability of the Morris water maze results without interference from physical activity variations (Figure 4c). During the probe trial, compared with the sham group, AD mice typically displayed longer durations and distances to locate the target platform. However, PQQ administration significantly reduced these extended durations and distances in AD mice (Figure 4d,f). Additionally, PQQ‐treated mice showed a preference for the target quadrant and displayed more direct paths to the target platform than the vehicle‐treated AD mice (Figure 4e,g). These findings indicate that PQQ effectively improves spatial learning and memory deficits in AD mice. Taken together, these behavioral tests emphasize the potential of PQQ in alleviating the cognitive dysfunctions associated with AD in mice.

### 2.6 Modulation of Synaptic Function by PQQ in AD Mouse Models

**Figure 5:**
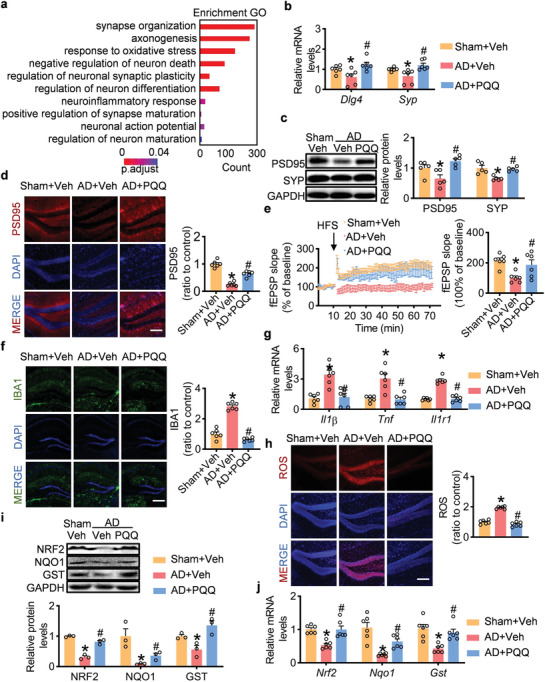
Synaptic protective benefits, anti‐inflammatory and antioxidant roles of pyrroloquinoline quinone (PQQ) in AD mouse. a) Gene Ontology (GO) enrichment analysis of differentially expressed genes. b) Quantitative assessment of relative mRNA expression levels for *Dlg4* and *Synaptophysin* (*n* = 6). c) Representative immunoblot images and subsequent statistical analysis of PSD95 and synaptophysin protein levels (*n* = 5). d) Fluorescence microscopy images and associated statistical analysis showcasing PSD95 expression in the hippocampus (scale bar = 200 µm, *n* = 6). e) Normalized fEPSP slope, benchmarked against the average fEPSP slope over a 10‐min pre‐high‐frequency stimulation baseline (*n* = 6). f) Representative fluorescence micrographs and statistical analysis of iBA1 in the hippocampus (scale bar = 1000 µm, *n* = 6). g) Relative mRNA levels of *Il1β*, *Tnf*, and *Il1r1*. (*n* = 6). h) Representative fluorescence micrographs and statistical analysis of DHE staining in the hippocampus (scale bar = 200 µm, *n* = 6). i) Representative bands and statistical analysis of NRF2, NQO1, and GST1 protein levels (*n* = 3). j) Relative mRNA levels of *Nrf2*, *Nqo1*, and *Gst1* (*n* = 6). Data are shown as mean ± SEM. ^*^ *p* < 0.05 versus control group; ^#^ *p* < 0.05 versus AD group.

To elucidate the mechanism underlying the protective effect of PQQ on cognitive dysfunction, we conducted an unbiased RNA sequencing (RNA‐seq) on brain tissue obtained from AD mice with and without PQQ administration (Figure S4a, Supporting Information). Gene ontology (GO) analysis of differentially expressed genes (DEGs) following PQQ administration in AD mice confirmed the neuroprotective role of PQQ, as evidenced by the increased expression of genes associated with synapse organization, axonogenesis, and anti‐neuronal death (**Figure** **5**a). Synaptic impairment and attrition are key characteristics of AD, closely linked with the deterioration of cognitive function.^[^ ^43^, ^44^ ^]^ Therapeutic strategies aimed at the rehabilitation of synaptic health and stability hold significant promise for influencing and regulating the activity of neural circuits.^[^ ^45^, ^46^ ^]^ Consequently, both mRNA and protein levels of postsynaptic density protein 95 (PSD95, encoded by Dlg4) and synaptophysin (SYP) were elevated after PQQ administration in AD mice (Figure 5b,c). Additionally, we observed an upregulation of PSD95‐positive signaling in both the hippocampus and cortex, suggesting that PQQ might counteract Aβ₁₋₄₂‐induced synaptic disruptions (Figure 5d; Figure S4b, Supporting Information). Long‐term potentiation plays a crucial role in modulating synaptic strength and is essential for memory formation and other cognitive functions. After high‐frequency stimulation, a significant reduction in the mean percent fEPSP slope was observed in Aβ₁₋₄₂‐treated mice. This impairment was resolved with PQQ administration, further emphasizing the capacity of PQQ to bolster synaptic function (Figure 5e). Our results indicate that PQQ offers neuroprotective effects in AD, particularly in mitigating synaptic dysfunction.

### 2.7 Anti‐Inflammatory and Antioxidant Roles of PQQ in AD Mouse Models

PQQ exerted specific effects on the expression of genes associated with anti‐neuroinflammatory and antioxidative stress responses, indicating its potential impact on inflammation and oxidative stress (Figure 5a). Given that significant inflammatory response occur within microglial cells in AD, we investigated the microglial state using immunofluorescence staining. Notably, PQQ treatment significantly suppressed microglial activation in both the hippocampus and cortex (Figure 5f; Figure S4c, Supporting Information). Moreover, a real‐time polymerase chain reaction (PCR) assay demonstrated decreased levels of proinflammatory cytokines Il1β and Tnfα, as well as the interleukin one receptor type 1 (Il1r1), a key mediator in several cytokine‐driven inflammatory pathways, following PQQ administration in AD (Figure 5g). These findings suggest a therapeutic role of PQQ in mitigating neuroinflammatory responses.

To investigate the antioxidative function of PQQ, dihydroethidium (DHE) staining was employed to detect the production of ROS. A notable reduction in ROS production was observed after PQQ administration (Figure 5h; Figure S4d, Supporting Information). Furthermore, PQQ enhanced the expression of antioxidant genes, including NAD(P)H: quinone oxidoreductase 1 (NQO1) and glutathione S‐transferase (GST), a pivotal oxidative stress mitigator, in AD mice. Additionally, PQQ administration increased the expression of NRF2, a central regulator of crucial antioxidative stress responses (Figure 5i,j). Taken together, these results suggest that the antioxidative effects of PQQ are mediated through the Nrf2 signaling axis.

### 2.8 PQQ‐Induced Activation of the SIRT1 Pathway

**Figure 6:**
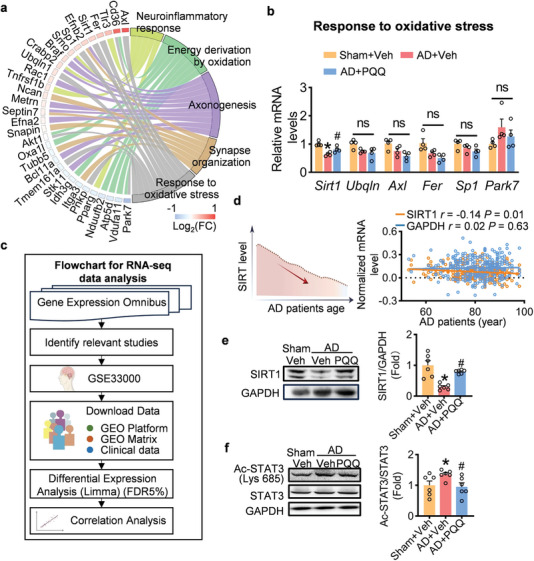
PQQ's Activation of the SIRT1 Pathway in AD. a) Gene Ontology (GO) enrichment analysis with a focus on functional categorization and specific genes associated with the identified GO terms. b) Relative mRNA levels of genes associated with antioxidative stress. (*n* = 4). c) Flowchart for AD patients’ RNA‐seq data analysis of prefrontal cortex (GSE33000). d) Correlation analysis highlighting the association between reduced Sirt1 mRNA levels and the age of AD patients. e) Representative bands and statistical analysis of SIRT1 protein levels (*n* = 6). f) Representative bands and statistical analysis of AC‐STAT3 and STAT3 protein levels (*n* = 6). Data are shown as mean ± SEM. ^*^ *p* < 0.05 versus control group; ^#^ *p* < 0.05 versus AD group.

Based on our initial findings, we delved into the molecular pathways modulated by PQQ in AD. SIRT1 emerged as a key gene with antioxidant properties within the AD model (**Figure** **6**a,b). To enhance the robustness of our model and mitigate any potential model‐specific bias, we expanded our analysis to include RNA‐sequencing data derived from the prefrontal cortex tissue of patients with AD, accessible in dataset GSE33000 (Figure 6c).

Importantly, a decrease in Sirt1 mRNA expression was correlated with the progression and severity of AD, suggesting a potential role for Sirt1 in the onset and development of AD (Figure 6d). Subsequently, we assessed the protein level of SIRT1 in the mouse cortex and observed an upregulation of SIRT1 after PQQ administration, indicating that PQQ facilitates the activation of the SIRT1 signaling pathway (Figure 6e). Furthermore, PQQ was found to bind to SIRT1, providing additional evidence for its ability to activate the SIRT1 pathway (Figure S5a, Supporting Information).

To validate the neuroprotective effect of PQQ via the SIRT1 signaling pathway, we examined the downstream target of SIRT1, namely, the signal transducer and activator of transcription 3 (STAT3), a crucial regulator of cerebral inflammation in neuroinflammation. Acetylated STAT3 enhances STAT3 phosphorylation, leading to the release of inflammatory mediators and amplification of the inflammatory cascade. We observed that PQQ abolished the AD‐induced increase in STAT3 acetylation (Figure 6f). These findings indicate that PQQ robustly activates the SIRT1 pathway in AD mice.

### 2.9 Inhibition of SIRT1 Negates the Neuroprotective Effects of PQQ

**Figure 7:**
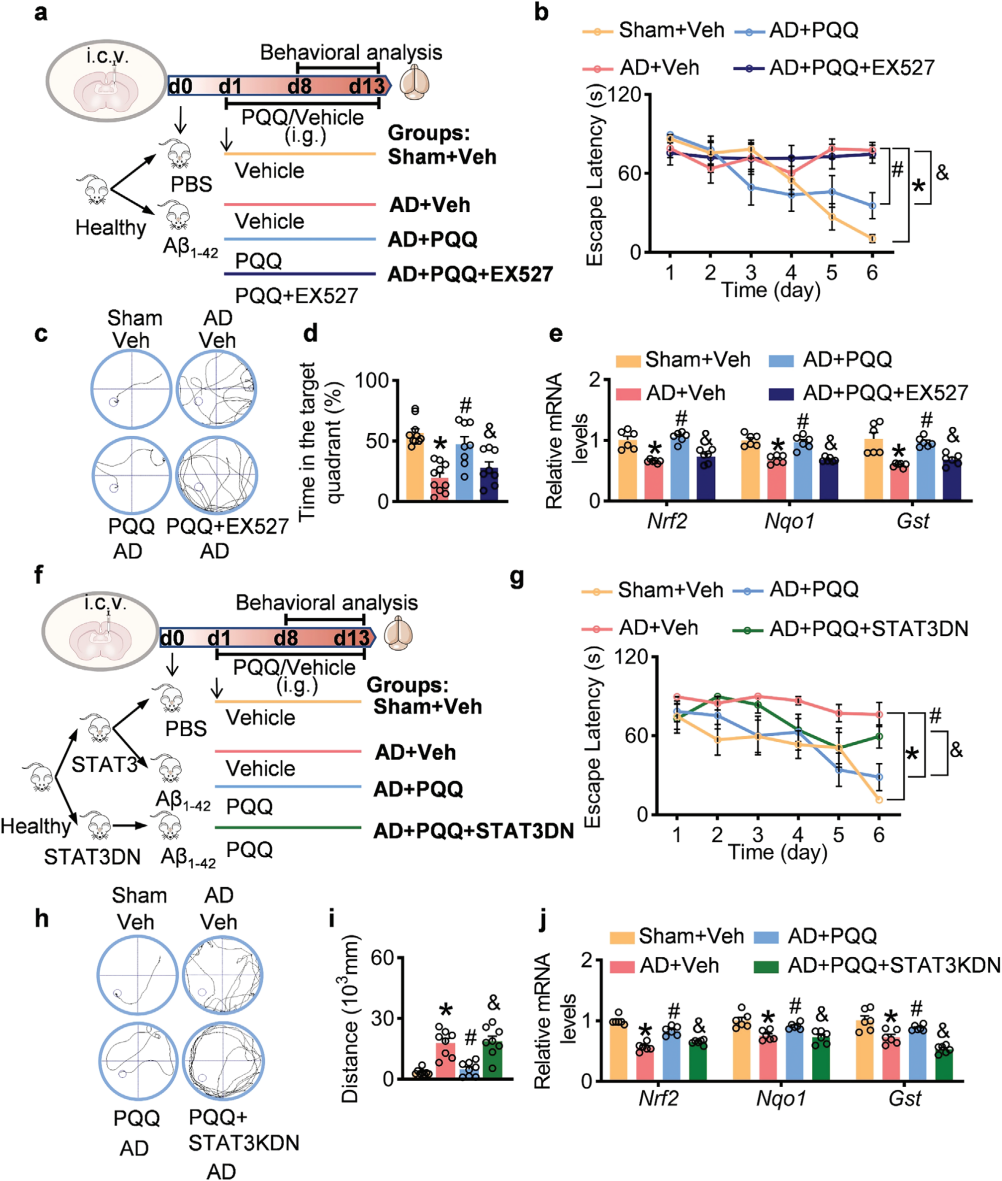
Impact of SIRT1 Inhibition and STAT3 Deacetylation on Neuroprotective Efficacy of pyrroloquinoline quinone (PQQ). a) Schematic of the experimental design (*n* = 10). b) Latency duration to locate the platform during the trial. c) Representative path tracings in the probe. d) The percentage of time spent in the target quadrant in the probe. e) Relative mRNA levels of NRF2, NQO1, GST1 (*n* = 6). f) Scheme of experimental design (*n* = 10). g) The latency to locate the platform during the experiment. h) Representative path tracings in the probe. i) The distance to reach the platform during the probe. j) Relative mRNA levels of *Nrf2*, *Nqo1*, *Gst1* (*n* = 6). Data are shown as mean ± SEM. ^*^ *p* < 0.05 versus control group; ^#^ *p* < 0.05 versus AD group; ^&^ *p* < 0.05 versus PQQ‐treated group.

To verify the role of SIRT1 in PQQ‐induced neuroprotection, we conducted an in vitro experiment on Aβ₁₋₄₂‐injured SH‐SY5Y cells using DHE staining. While PQQ effectively countered the ROS production induced by Aβ in SH‐SY5Y cells, the SIRT1 inhibitor EX527 reduced the ability of PQQ to suppress ROS (Figure S5b, Supporting Information). In vivo, EX527 was administered to PQQ‐treated AD mice (**Figure** **7**a). Behavioral analyses using the Morris water maze showed that mice treated with both PQQ and EX527 displayed impaired navigational abilities. This was evident from the increased time taken to locate the target platform, decreased time spent in the target quadrant, and the longer distances traveled to reach the platform compared with those of the group treated only with PQQ (Figure 7b–d).

Contrary to the PQQ‐only group, the administration of EX527 negated the upregulation of *Nrf2*, *Nqo1*, and *Gst* induced by PQQ (Figure 7e). Furthermore, DHE staining of brain sections revealed that EX527 reduced the antioxidative benefits conferred by PQQ in the Aβ₁₋₄₂‐induced AD mouse model (Figure S5c,d, Supporting Information). The treatment with EX527 also suppressed the PQQ‐induced activation of the SIRT1 pathway (Figure S5e, Supporting Information). Additionally, EX527 administration blocked the beneficial function of PQQ on synaptic modulation and neuroinflammation (Figures S5f–h and S6a,b, Supporting Information). Taken together, these findings highlight the crucial role of the SIRT1 pathway in facilitating the neuroprotective effects of PQQ in AD.

### 2.10 Deacetylation of STAT3 Impairs the Neuroprotective Role of PQQ in AD

Considering that the transcriptional activity of STAT3 occurs after its acetylation, we administered a STAT3 dominant negative (STAT3DN) variant with lost deacetylation ability at K679, K685, K707, and K709 (K679QK685QK707QK709Q) to PQQ‐treated AD mice using an adenovirus delivery system (Figure 7f). While PQQ decreased the escape latency and distance in AD mice, STAT3DN abolished the therapeutic efficacy of PQQ (Figure 7g–i). Furthermore, the expression of STAT3DN attenuated the antioxidative response to PQQ, manifesting as suppressed *Nrf2*, *Nqo1*, and *Gst* mRNA levels and elevated ROS accumulation in PQQ‐treated AD mice (Figure 7j; Figure S7a,b, Supporting Information). STAT3DN further interfered with PQQ‐driven synaptic improvement and reduction of neuroinflammatory marker levels in mice with AD (Figures S7c–e and S8a,b, Supporting Information). These results suggest that obstructing STAT3 acetylation inhibits the recovery of neuroprotective effects provided by PQQ.

### 2.11 Modulation of the CREB Pathway by PQQ in AD

**Figure 8:**
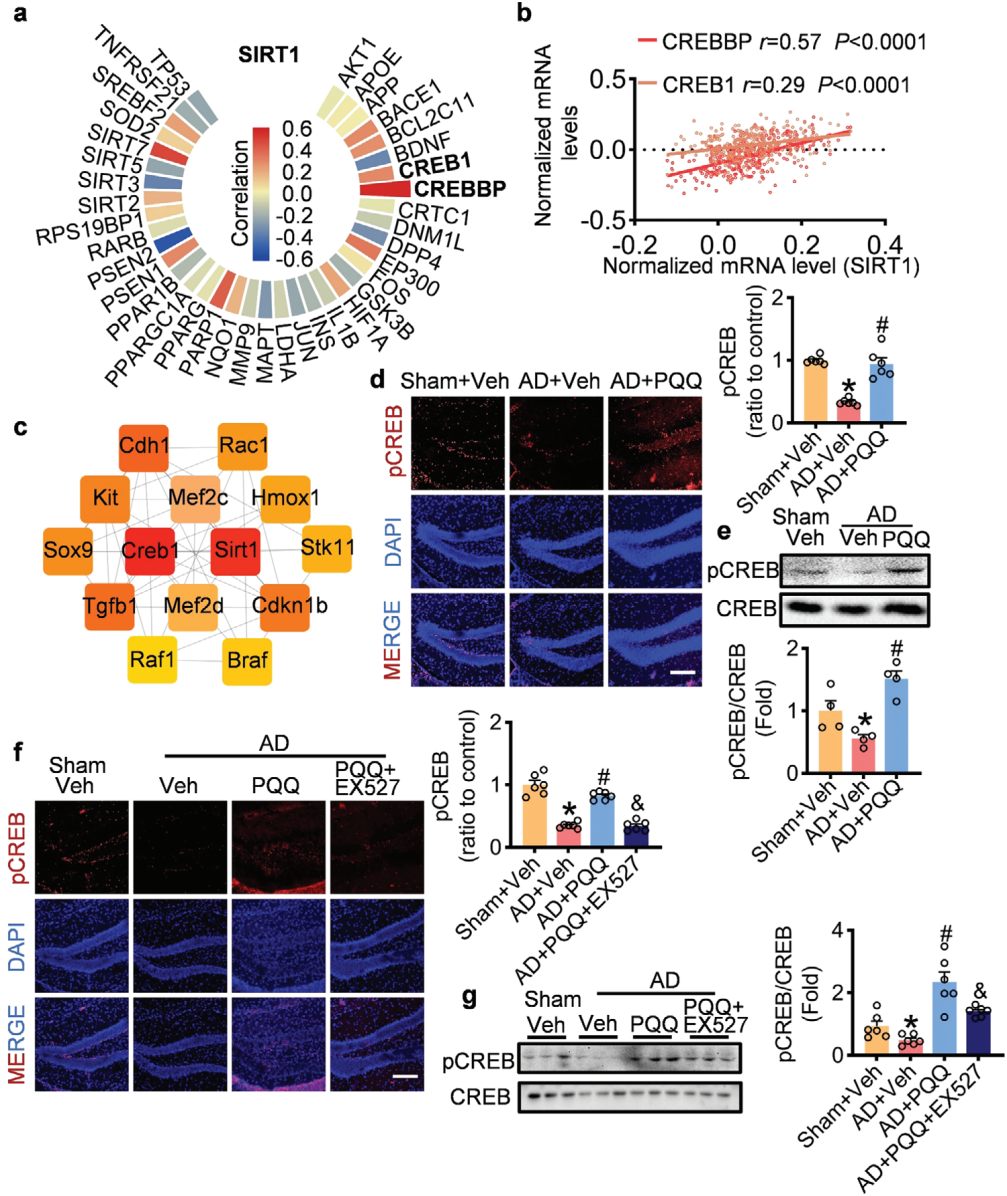
PQQ's regulatory influence on the CREB signaling pathway in AD. a) Analysis showcasing the correlation between SIRT1 and its associated proteins in AD patients. b) Depiction of the correlation between CREBBP, CREB1, and SIRT1. c) Key genes pinpointed from the protein‐protein interaction network utilizing the Maximal Clique Centrality algorithm in CytoHubba. d) Representative fluorescence micrographs and statistical analysis of pCREB in the hippocampus (scale bar = 200 µm, *n* = 6). e) Representative bands of pCREB and CREB and statistical analysis of pCREB/CREB expression levels (*n* = 4). f) Representative fluorescence micrographs and statistical analysis of pCREB in the hippocampus (scale bar = 200 µm, *n* = 6). g) Representative bands of pCREB and statistical analysis of pCREB (*n* = 6). Data are shown as mean ± SEM. ^*^ *p* < 0.05 versus control group; ^#^ *p* < 0.05 versus AD group. ^&^ *p* < 0.05 versus the PQQ‐treated group.

Our data revealed that PQQ has a modulatory effect on gene expression profiles. Through further validation, we confirmed that specific genes are regulated through SIRT1‐mediated pathways following PQQ treatment. While some genes are influenced by the SIRT1 signaling pathway upon PQQ administration, others remain unaffected by SIRT1. Recognizing that PQQ operates through intricate signaling pathways to protect against AD progression and identifies SIRT1 as a crucial node in this network, we aimed to investigate if other factors contribute to SIRT1‐mediated neuroprotection after PQQ administration. To accomplish this, we analyzed previous datasets to explore the relationship between SIRT1 and its interacting proteins in AD patients. Our analysis revealed associations between SIRT1 and both CREB‐binding protein (CREBBP) (*r* = 0.57, *p* < 0.001) and cyclic adenosine monophosphate (cAMP) response element‐binding protein 1 (CREB1) (*r* = 0.30, *p* < 0.001) (**Figure** **8**a,b). CytoHubba's Maximal Clique Centrality algorithm further highlighted CREB1 as a central component of the PQQ regulatory network (Figure 8c).

To investigate the interaction between the CREB pathway and the neuroprotective effects of PQQ, we conducted a series of molecular assays. Immunofluorescence assays demonstrated enhanced CREB activation in the hippocampus and cortex following PQQ treatment (Figure 8d; Figure S7a, Supporting Information). Western blotting confirmed a significant increase in CREB phosphorylation and activation in the presence of PQQ (Figure 8e). Importantly, we found that the SIRT1 inhibitor EX527 attenuated the PQQ‐induced CREB activation (Figure 8f,g; Figure S7b, Supporting Information).

**Figure 9:**
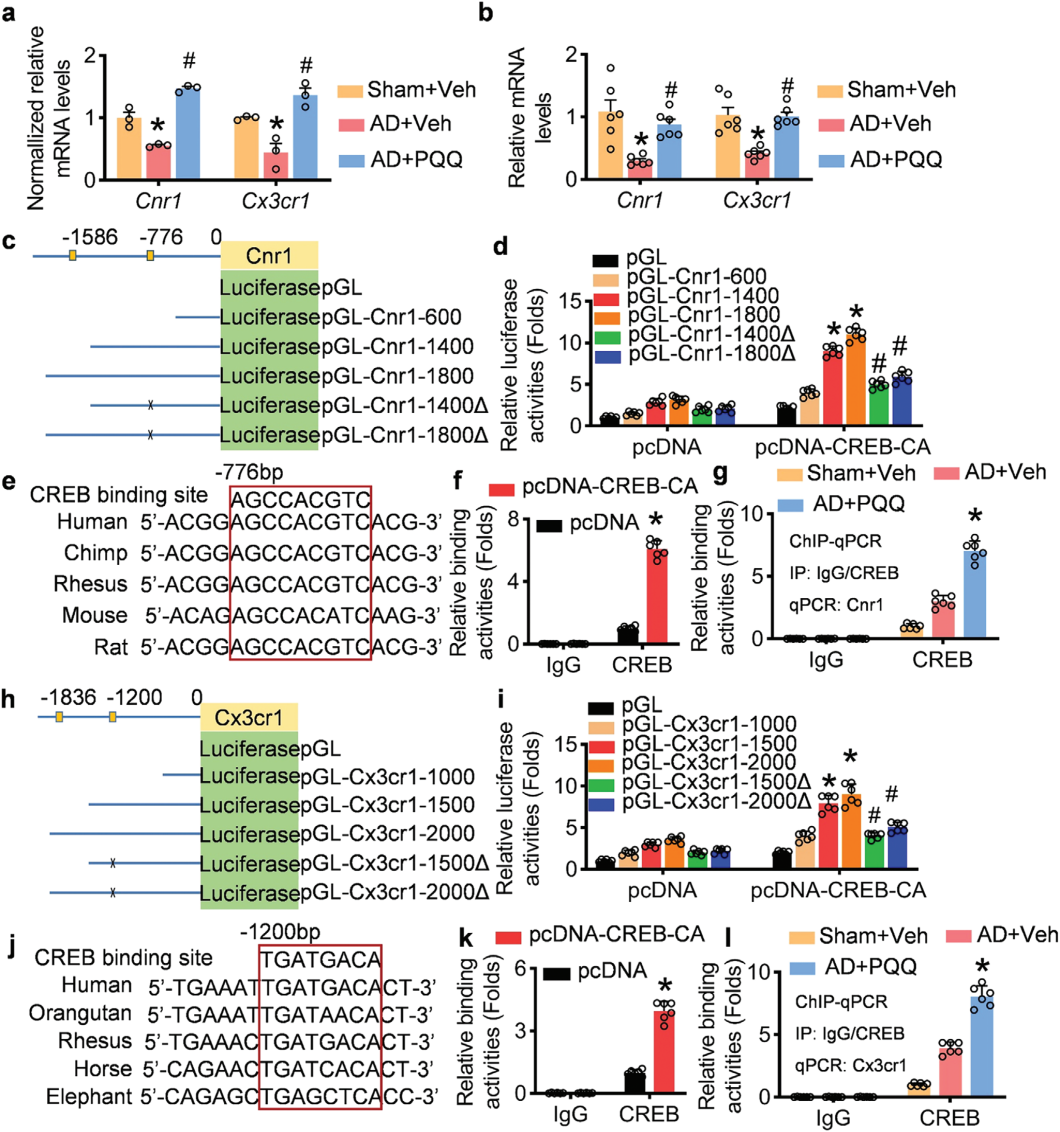
CREB promotes pyrroloquinoline quinone (PQQ)‐induced upregulation of Cnr1 and Cx3cr1 expression. a) mRNA levels of *Cnr1* and *Cx3cr1*, normalized to internal controls. b) Relative mRNA levels of *Cnr1*and *Cx3cr1* (*n* = 6). c) Schematic of potential CREB‐binding sites on the Cnr1 promoter and the corresponding reporter constructs for luciferase assays. d) Luciferase activity corresponding to various lengths of the Cnr1 promoter (*n* = 6). e) Conservation analysis of the Cnr1 promoter region at −776 bp across five mammalian species. f,g) ChIP‐qPCR analysis demonstrating CREB's association with the Cnr1 promoter in HEK293 cells and brain tissues, respectively (*n* = 6). h) Schematic of putative CREB‐binding sites on the Cx3cr1 promoter and associated reporter constructs for luciferase assays. i) The luciferase activities of luciferase reporter plasmids containing different lengths of the Cx3cr1 promoter (*n* = 6). j) The Cx3cr1 promoter located at −1200 bp upstream of the transcription start site, is conserved among five mammalian species. k,l) ChIP‐qPCR analysis demonstrating CREB's association with the Cx3cr1 promoter in HEK293 cells and brain tissues, respectively (*n* = 6). Data are shown as mean ± SEM. ^*^ *p* < 0.05 versus control group; ^#^ *p* < 0.05 versus AD group.

Altered CREB‐mediated transcriptional activity has been observed in AD and may contribute to the cognitive impairments associated with the disease. To investigate the downstream effects of CREB1 in AD post‐PQQ administration, we performed a real‐time PCR assay based on RNA‐seq results, confirming the upregulation of cannabinoid receptor 1 (Cnr1) and CX3C chemokine receptor 1 (Cx3cr1) expression by PQQ (**Figure** **9**a,b). Using the transcriptional factor prediction software JASPER, we analyzed the Cnr1 promoter and identified two CREB1 binding sites at −1536 and −776 bp (Figure 9c). Luciferase assays demonstrated increased luciferase activity in both pGL‐Cnr1‐1400 and pGL‐Cnr1‐1800 compared with pGL‐Cnr1‐600, which lacks the sequence at −1536 bp (Figure 9d). Removing this site (−1536 bp) reduced luciferase activity relative to pGL‐Cnr1‐1400 and pGL‐Cnr1‐1800, indicating that CREB1 can bind to the Cnr1 promoter at −1536 bp (Figure 9d). This site exhibits conservation across species, underscoring its importance for Cnr1 functionality (Figure 9e). In HEK293 cells, CREB1 overexpression increased the binding between CREB1 and the Cnr1 promoter (Figure 9f). Chromatin immunoprecipitation (ChIP) assays were performed to assess if PQQ enhances CREB1 binding affinity to the Cnr1 promoter (Figure 9g).

Additionally, JASPER predicted two CREB1 sites on the Cx3cr1 promoter (Figure 9h). Luciferase assays confirmed the proximity of CREB1 to the Cx3cr1 promoter at −1200 bp, another highly conserved site across species (Figure 9i,j). CREB1 overexpression enhanced CREB1 binding to the Cx3cr1 promoter, and this process was further enhanced by PQQ (Figure 9k,l). These findings elucidate that PQQ administration not only increases the expression of these genes but also augments their regulation via the CREB signaling pathway.

## 3 Discussion

Exploring effective anti‐AD drugs is both laborious and costly. These challenges are exacerbated by the prolonged duration and intricacy of AD drug discovery. In this study, we employed deep learning to expedite the screening process. This method boosts efficiency, narrows the screening range, and mitigates the conventionally labor‐intensive nature of anti‐AD drug research. Through the fusion of deep learning and meta‐analysis, we identified PQQ from food sources as a potent, low‐toxicity compound appropriate for daily intake. We delved into the molecular mechanism of PQQ as a promising therapeutic contender. Importantly, our data revealed that AD patients had markedly reduced peripheral blood PQQ levels compared with healthy individuals, aligning with our deep learning and meta‐analysis insights. Additionally, a decline in PQQ levels corresponded with the progression of cognitive impairment, intimating the potential protective ability of PQQ in cognitive health.

The deep learning technique used in this research augments the role of artificial intelligence (AI) in pharmacological studies, especially in the field of anti‐AD drug discovery. The model was meticulously trained on an extensive dataset of molecular structures, equipping it to accurately predict the BBB permeability, as well as the anti‐inflammatory and antioxidative attributes of various compounds. This precision reduces the need for experimental validation of unsuitable compounds, ensuring a focused approach to pinpointing compounds with robust therapeutic potential and enhancing resource efficiency throughout the drug discovery journey.

A standout feature of our method is the development of a holistic platform that seamlessly combines anti‐inflammatory, antioxidant, and BBB permeability characteristics into a single model for neuropharmacological drug screening, with an emphasis on AD. Built on a solid foundation of molecular structures and underpinned by a sophisticated grasp of neuropharmacology, this platform was devised to discern compounds manifesting a trio of attributes vital for effective neurological drug function. The model's ability to assess these three key characteristics showcases an innovative approach to AI‐aided drug discovery, offering a comprehensive, targeted, and resource‐efficient strategy for uncovering potential AD treatments. This integration enhances the accuracy of recognizing compounds with a strong therapeutic potential. This research paves the way for a fresh perspective on deep learning's application in neuropharmacological exploration, specifically in AD drug discovery.

Considering that AD is a multifactorial disease, PQQ may influence several facets of AD progression. The intertwined roles of neuroinflammation and oxidative stress in the etiology of AD are well‐established. Our data spotlight PQQ's capability to counteract both processes. The significant decrease in proinflammatory cytokines and subdued microglial activation after PQQ administration point to promising avenues for AD management. The oxidative response is also suppressed in AD patients treated with PQQ. PQQ mitigates ROS accumulation and boosts the expression of antioxidant genes. Due to PQQ's multifaceted neuroprotective effects, it emerges as a potential therapeutic strategy for AD patients and those at elevated risk for AD.

The signaling pathways tied to PQQ‐mediated neuroprotection are intricate. The principal targets of PQQ in AD are SIRT1 and CREB1, central players in PQQ‐related pathways. SIRT1 activation influences downstream targets, manages oxidative stress, delivers neuroprotective and anti‐neuroinflammatory outcomes, and thus stands out as a vital modulator of AD pathogenesis.^[^ ^47^ ^]^ A key downstream target of SIRT1 is STAT3, which undergoes deacetylation via SIRT1‐mediated modulation. PQQ induced SIRT1 activity and deacetylated STAT3. CREB1, another central gene for PQQ in AD, is noteworthy. The cyclic adenosine monophosphate (cAMP) response element‐binding protein (CREB) is an essential transcription factor involved in numerous cellular activities, including synaptic plasticity, neuroprotection, and memory consolidation.^[^ ^48^ ^]^ Post‐mortem analyses of AD‐affected brains have showcased reduced CREB phosphorylation, highlighting the disrupted functional landscape of the disease environment.^[^ ^49^ ^]^ PQQ amplifies the transcriptional activity of CREB1 in AD, raising the expression of the anti‐inflammatory genes Cnr1 and Cx3cr1.

**Figure 10:**
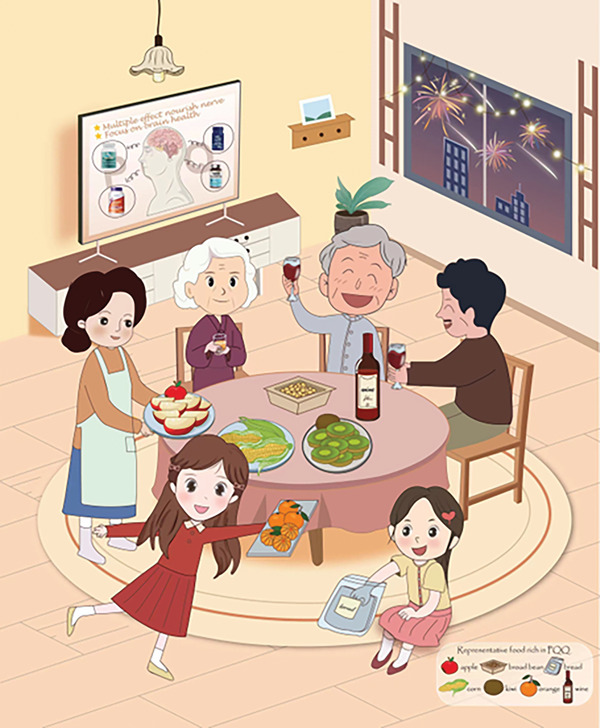
PQQ: A potential therapeutic candidate with efficacy and minimal toxicity for Alzheimer's disease management. Illustration highlighting the benefits of pyrroloquinoline quinone‐enriched diets in Alzheimer's disease management.

In conclusion, this study marks a significant amalgamation of deep learning and pharmacological research, accentuating the therapeutic promise of PQQ as an agent against AD. Through the integration of interdisciplinary fields, the drug discovery process is accelerated, increasing precision in targeting disease mechanisms and improving resource efficiency. Our findings advocate for the consideration of PQQ‐centric interventions, which may include dietary modifications to incorporate PQQ‐rich foods as a proactive approach to AD management. The neuroprotective efficacy of PQQ, as revealed in **Figure** **10**, intimates that a diet augmented with PQQ sources—such as apples, natto, bread, corn, kiwi fruits, and oranges—might confer tangible benefits in delaying or alleviating the clinical manifestation of AD. Therefore, the incorporation of these nutritive elements into the diets of individuals at heightened risk of AD could be a viable strategy to delay disease progression. Prospective nutritional guidelines should recommend PQQ intake to enhance cognitive health. The insights from this research provide firm groundwork for further research into the medicinal benefits of PQQ and outline a cohesive strategy for merging deep learning with pharmacology in the advancement of drug research and development.

## 4 Experimental Section

### Deep Learning Methodology for Compound Analysis—Data Collection

The compound library of the BBB was derived from a previous study.^[^ ^50^ ^]^ The anti‐inflammatory‐related pathways (WP:3844, GO:0000165, GO:0 004896, KEGG: hsa04064, KEGG: hsa04210, KEGG: hsa04630) and antioxidant related pathways (GO:00 98869, GO:00 72593, GO:0 006281) were searched in the Kyoto Encyclopedia of Genes and Genomes (KEGG), GO, WikiPathways databases. Then, STRING was used to perform protein–protein interaction analysis on the genes in each pathway, and Cytoscape was used to calculate the key genes of each pathway. BioAssays for the key genes were subsequently searched on PUBCHEM, which is a database containing activity test results for specific genes (activity test results of compounds are usually labeled active, inactive, inconclusive, etc.). Active compounds were labeled as 1, which was expected to exert anti‐inflammatory or antioxidative effects by affecting a specific pathway; inactive were marked as 0, which did not produce anti‐inflammatory or antioxidant effects from these specific pathways; and inconclusive compounds were not included in the data set due to unknown activity. Finally, a total of 11 542 compounds were collected, with nine attributes.

### Deep Learning Methodology for Compound Analysis—Model Selection Rationale

The selection of modeling methodologies was influenced by preliminary experiments. While the MLP variant model, implemented using Fastai's tabular learner,^[^ ^51^ ^]^ was initially applied across all properties, its performance varied. The model notably excelled for the BBB and anti‐inflammatory properties but was less effective for the antioxidative properties. On the other hand, the GCN model, implemented using deepchem's GCNModel, did not yield optimal results for BBB and anti‐inflammatory properties. The initial foray into multi‐task learning did not result in enhanced performance. Instead of leveraging shared features across properties, this approach led to diminished results. Hence, individual models were designed for each property. For optimizing model configurations, Optuna,^[^ ^52^ ^]^ an automated hyperparameter tuning tool, resulting in 10 distinct models, each optimized for specific properties (Table S1, Supporting Information), was employed.

### Deep Learning Methodology for Compound Analysis—Dataset Processing and Feature Extraction

- *For the MLP variant*: Initially, the dataset consisted of 11 542 compounds. However, due to RDKit's limitations in processing salts, 18 compounds were excluded, leaving 11 524 compounds. Using RDKit, 12 molecular descriptors, based on empirical rationale, were extracted:

**“ExactMolWt”**: Represents the exact molecular weight of the compound.

**“MolLogP”**: LogP value, indicating the lipophilicity of the compound. Lipophilicity often relates to a drug's ability to cross the BBB.

**“TPSA”**: Total polar surface area, which can provide insights into a molecule's membrane permeability.

**“NumHDonors”**: The number of hydrogen bond donors in the molecule.

**“NumHAcceptors”**: The number of hydrogen bond acceptors in the molecule.

**“NumRotatableBonds”**: Gives an idea of the molecule's flexibility.

**“FractionCSP3”**: Describes the molecule's sp3 character, which can be a measure of its three‐dimensionality.

**“NumAromaticRings”**: The number of aromatic rings present in the molecule.

**“MaxPartialCharge”**: The maximum partial charge present in the molecule.

**“MinPartialCharge”**: The minimum partial charge present in the molecule.

**“NumNitrogen”**: The number of nitrogen atoms in the molecule.

**“NumOxygen”**: The number of oxygen atoms in the molecule.

- *For the GCN Model*: The original dataset consisting of 11 542 compounds, including those containing salts, was processed. Feature extraction was performed using deepchem's MolGraphConvFeaturizer. This process involved the encoding of atomic properties (such as atom type, charge, and hybridization) and bond characteristics (such as bond type and conjugation status). Each atom and bond and represented by vectors of length 30 and 11, respectively. For a comprehensive description of the feature extraction process, readers are referred to [https://deepchem.readthedocs.io/en/stable/api_reference/featurizers.html#molgraphconvfeaturizer].

### Deep Learning Methodology for Compound Analysis—Addressing Data Imbalance

For the MLP model, given the initial dataset of 11 542 compounds, inherent data imbalances (Table S2, Supporting Information) prompted the introduction of the FocalLoss, with its parameters optimized via Optuna. Despite leveraging stratified train‐validation‐test splits, performance improvements were sought, which led to the incorporation of oversampling techniques including SMOTE^[^ ^53^ ^]^ and ADASYN,^[^ ^54^ ^]^ guided by Optuna. This approach notably enhanced model performance.

For the GCN model, challenges with conventional oversampling techniques arose from various aspects. Techniques such as SMOTE were found unsuitable for generating valid molecular graphs. Additionally, simple repetitions of minority class samples risked overfitting. Given these challenges, the majority class was randomly downsampled. As a result, the dataset for the antioxidative property GO:00 98869 comprised 2118 compounds, for GO:0 006281 it was 2682 compounds, and for GO:00 72593, the dataset had 2296 compounds.

### Deep Learning Methodology for Compound Analysis—Model Training and Evaluation

Both models were meticulously evaluated using five metrics: accuracy, precision, recall, F1 score, and ROC‐AUC. Special emphasis was placed on the F1 score due to the highly imbalanced nature of the data, as detailed in Table S2 (Supporting Information).

- *For the MLP Model*: Given the inherent imbalances in the dataset, oversampling techniques, such as SMOTE and ADASYN, were applied. After achieving a balanced dataset, data were partitioned by reserving 20% for testing. From the remaining 80%, another 20% was set aside for validation, with the rest dedicated to training.
- *For the GCN Model, the Training Process Was Threefold*: Due to the data imbalances, the majority class was randomly downsampled. With the balanced dataset, a fivefold cross‐validation approach was adopted to determine the optimal hyperparameters and model configuration, ensuring robust performance across the different folds. After identifying the best model parameters through cross‐validation, the model was trained on the entire training dataset to maximize its potential performance. This fully trained model was subsequently used for predicting the outcomes of the target attributes.

### Deep Learning Methodology for Compound Analysis—t‐SNE Visualization of Compound Features

- *Feature Extraction*: MLP Model: For the compounds processed by the MLP model, 12 molecular descriptors were extracted using the RDKit library. These descriptors served as the high‐dimensional features that were subsequently used for *t*‐SNE visualization.
- GCN Model: For the compounds processed by the GCN model, molecular graph features were obtained using the MolGraphConvFeaturizer from deepchem. After feature extraction, a mean pooling strategy was employed to aggregate node features, creating a fixed‐size vector for each compound.
- *t‐SNE Parameters and Visualization*: The *t*‐SNE plots were generated using a perplexity of 30 and a maximum number of iterations set to 1000.

To distinguish specific sets of compounds in visualization, those ultimately selected based on their scores and non‐toxicity were marked as “top hits” in the plots.

### Antibodies, Reagents, and Adenoviruses

The antibodies used are listed in Table S5 (Supporting Information). Recombinant human Aβ_1–42_ peptide was procured from Beyotime Biotechnology (China). Recombinant adenoviruses, encompassing STAT3 wild‐type and K679QK685QK707QK709Q mutants, were meticulously constructed and packaged by Genechem Co., Ltd. (Shanghai, China).

### Systematic Review and Meta‐Analysis

This systematic review and meta‐analysis adhered to the guidelines stipulated in the Preferred Reporting Items for Systematic Reviews and Meta‐Analyses statement.^[^ ^55^ ^]^ A comprehensive protocol was designed a priori and subsequently registered with PROSPERO under the registration number PROSPERO CRD42023430260. The commencement of this endeavor involved a systematic exploration of multiple databases, including PubMed, Embase, Wiley, and Web of Science, conducted on May 20, 2023. Additionally, an exhaustive examination of the references cited within the included articles was conducted.

The inclusion criteria focused on cohort studies that assessed the associations between adherence to the MIND diet and the incident of dementia or AD. Subsequently, pertinent data elements were meticulously extracted, including the identity of the primary author, year of publication, cohort designation, geographical location, sample size, baseline age range, methods employed for dietary assessment, approaches utilized for dementia ascertainment, as well as risk estimates and their respective 95% confidence intervals, derived from multivariable‐adjusted models, alongside the incorporated covariates. Study quality was evaluated through the rigorous application of the Newcastle‐Ottawa scale.

To ensure the highest standards of reliability and accuracy, two independent reviewers, namely, S.L. and L.C., conducted the initial literature screening, data extraction, and risk of bias assessment. Instances of disagreement or discordance were judiciously resolved through consultation with the third author, J.L.

For the synthesis of findings, common‐effect models were employed to amalgamate risk estimates comparing the highest versus the lowest levels of MIND diet adherence. The statistical heterogeneity across studies was quantified using the I^2^ statistic, with data analysis facilitated through the utilization of the R package “meta” from the R Project for Statistical Computing. In accordance with the guidelines delineated in the Cochrane Handbook, an I^2^ statistic of ≤40% was indicative of minor heterogeneity, while values within the range of 30–60% denoted moderate heterogeneity. Substantial heterogeneity was represented by I^2^ values of 50–90%, and I^2^ values of >75% signified considerable heterogeneity. Furthermore, the presence of publication bias was assessed using the Egger test.^[^ ^56^ ^]^

### Quantification of PQQ in Human Serum

All participants provided written informed consent, and the study protocol, including recruitment strategies and ethical considerations, was approved by the Institutional Review Board (IRB) of Affiliated Brain Hospital of Nanjing Medical University with the number 2020‐KY043‐01. The specific information of all patients is summarized in Table S6 (Supporting Information).

Serum samples (100 µL) underwent a meticulous sample preparation procedure, commencing with the addition of 100 µL of 50% methanol and 200 µL of acetonitrile. The resultant mixture was subjected to vigorous vortexing for a duration of 5 min and subsequently underwent centrifugation at 20 000 g for 10 min. The supernatant, meticulously handled, was then transferred into a vial. A precise 2 µL aliquot of this supernatant was introduced into the state‐of‐the‐art ultra‐performance liquid chromatography‐electrospray ionization‐tandem mass spectrometry system for further analyses.

Chromatographic separation was performed using the highly sophisticated Acquity UPLC‐I‐Class System, manufactured by Waters (Milford, MA, USA). This system was equipped with a reversed‐phase column (BEH C18, dimensions: 2.1 mm × 50 mm, particle size: 1.7 µm; sourced from Waters) and maintained at a constant temperature of 35 °C throughout the analytical process. The flow rate was meticulously set at 0.3 mL min^−1^. The mobile phase included a binary solvent system, comprising 5 mm ammonium formate in water (referred to as A) and acetonitrile (referred to as B). The gradient elution scheme employed for the separation was as follows: 0–0.5 min, 25% B; 0.5–2.5 min, a linear increase from 25% to 95% B; 2.5–3.5 min, 95% B; 3.5–3.6 min, a linear decrease from 95% to 25% B; 3.6–6 min, 25% B.

The analytical component of this study involved the utilization of the Xevo TQ‐S micro‐Triple‐Quadrupole Tandem Mass Spectrometer, also manufactured by Waters. An electrospray ionization source, operating in the negative ionization mode, was employed for mass spectrometric analysis. Quantification of PQQ was achieved using the multiple reaction monitoring mode, with specific transitions of m/z 329.16 to 241.14 being monitored. Ionization parameters were meticulously adjusted, with a cone voltage set at 20 V and a collision energy maintained at 12 eV. Additional instrumental settings included a capillary voltage of 2500 V, a source temperature of 120 °C, a desolvation temperature of 400 °C, a desolvation gas (nitrogen) flow rate of 600 L h^−1^, and a cone gas (nitrogen) flow rate of 25 L h^−1^.

The wealth of data generated was subjected to comprehensive processing and analysis utilizing Masslynx software, specifically version 4.2, developed by Waters (Manchester, UK). This software played an integral role in facilitating data interpretation and subsequent elucidation of results.

### Mice and Drug Intervention

All male C57/BL6 mice (8 weeks old, weight 18–22 g) were obtained from the Changzhou Cavens Laboratory Animal Company. Animal culture and procedures were approved by the Pharmaceutical Laboratory Animal Center of China Pharmaceutical University with the number 2023‐08‐005. To induce the formation of oligomeric Aβ_1–42_, the Aβ_1–42_ peptide was dissolved in sterile phosphate‐buffered saline (PBS) at a concentration of 2 mg mL^−1^ and incubated at 37 °C for 24 h. Subsequently, the AD mouse model was established by the intracerebral injection of 5 µL of the oligomerized Aβ_1–42_ peptide into the lateral ventricle on the first day of the experiment. The injection point with a brain stereo‐positioning instrument: −1.0 ± 0.06 mm posterior to bregma, 1.8 ± 0.1 mm lateral to the sagittal suture, and 2.4 mm in depth.

For the control group, an equivalent volume of sterile saline was administered in a similar manner. Following the Aβ_1–42_ injection, a 24 h interval was observed, after which mice received intragastric administration of 20 mg kg^−1^ of PQQ for a duration spanning from day 2 to day 14 of the experiment. The selection of PQQ administration timing and dosage was informed by a meticulous review of the literature.^[^ ^57^, ^58^ ^]^ Control and model group mice were exclusively subjected to intragastric gavage with normal saline. To further validate the potential protective mechanism mediated through the SIRT1 pathway, some mice were treated with PQQ (20 mg kg^−1^, intragastric) in combination with EX527 (2 mg kg^−1^, intragastric). To elucidate the role of STAT3 deacetylation, another subset of mice received lateral ventricle injections of recombinant adenoviruses carrying either wild‐type STAT3 or mutants bearing K679QK685QK707QK709Q substitutions. Following adenovirus induction, these mice were subjected to Aβ_1–42_ injections and received subsequent administration of 20 mg kg^−1^ PQQ.

### Cell Culture and Treatment

The SH‐SY5Y cell line, sourced from Shanghai Zhongqiaoxinzhou Company in Shanghai, China, and the HEK293 cell line, obtained from Guangzhou Jennio Biotech in Guangzhou, China, were cultured in Dulbecco's modified Eagle medium (obtained from Invitrogen). The culture medium was supplemented with 10% fetal bovine serum and 1% penicillin (100 units mL^−1^)/streptomycin (100 mg mL^−1^). Cell cultures were maintained in a controlled environment with a humidity level of 5% CO_2_ and a temperature of 37 °C.

### Behavioral Analysis

The Y‐maze served as the primary tool for assessing spatial working memory in rodents. This maze structure was constructed as a symmetrical Y‐shaped apparatus, featuring three identical arms evenly spaced at 120° apart. Crafted from black plexiglass, the maze provided a controlled environment for experimentation. During evaluation, mice were gently placed into one of the arms and given a 10‐min window to freely explore the entire maze. Detailed records were maintained of the sequence and frequency with which the mice entered each arm. In the context of this assessment, normal mice typically exhibited a high degree of spontaneous alternating behavior, characterized by successive visits to different open arms (e.g., A–B–C–A–B). The number of correct alternating responses was tallied, and the spontaneous alternating rate was subsequently computed.

The Morris water maze was another integral tool employed for evaluating the spatial learning and memory capabilities of mice. This comprehensive maze system consisted of a large pool housing an underwater platform. The pool had a radius of 60 cm and a height of 45 cm and was divided into four quadrants in a randomized fashion. The submerged platform, positioned just 1 cm beneath the water surface, was situated at the center of one of the quadrants. To render the water opaque, titanium dioxide was added, and the water temperature was meticulously maintained at ≈25 °C. Behavioral experiments were initiated 7 days after the commencement of drug administration, encompassing a 5‐day training phase and spatial learning and memory assessments on the sixth day. During the daily training trials, mice were placed in the pool, facing the pool wall, and were tasked with locating the submerged platform positioned 1 cm above the water's surface. If a mouse successfully reached the platform within a 90 s time frame, it was permitted to rest on the platform for 10 s. Conversely, if a mouse failed to locate the platform within the designated time, it was manually guided to the platform and allowed to rest there for 10 s to facilitate memory of the platform's location. On the sixth day of the experiment, the platform was submerged 1 cm beneath the water's surface, and a spatial exploration trial was conducted. During all phases of testing, various parameters, including latency, path length, swimming velocity, residence time in the target quadrant, and the trajectory traversed, were meticulously recorded by a computerized system.

### Western Blot

On day 14, mice were humanely euthanized under deep anesthesia, and to ensure complete perfusion, cold PBS was pumped through the left ventricle at a controlled flow rate of 10 mL min^−1^ for a duration of 60 s. Following cardiac perfusion, the brains were meticulously extracted, and total cellular proteins were extracted from the cerebral cortex tissues. This was done by lysing the tissues with RIPA buffer (procured from Beyotime) supplemented with phenylmethylsulfonyl fluoride. Afterward, the lysates underwent centrifugation at 12 000 rpm min^−1^ at 4 °C for 30 min to collect the supernatant. The quantification of protein content was performed using a BCA kit (also obtained from Beyotime).

The isolated proteins were subjected to electrophoresis on SDS‐PAGE (sodium dodecyl sulfate‐polyacrylamide gel electrophoresis) and subsequently transferred onto polyvinylidene difluoride fluoride (PVDF) membranes, sourced from Millipore. Following the transfer, the membranes were subjected to blocking with 5% skim milk, prepared in Tris‐buffered saline containing 0.1% Tween20 (TBST), for a duration of 1 h at room temperature. Subsequently, the membranes were incubated overnight at 4 °C with primary antibodies, suitably diluted in a blocking solution.

After thorough washing with TBST for five cycles, the membranes were then subjected to incubation with secondary antibodies (either HRP‐conjugated anti‐rabbit IgG or HRP‐conjugated anti‐mouse IgG) for 1 h at room temperature. Detection of protein expression was accomplished using an enhanced chemiluminescence kit (Tanon) and visualized using a Gel Imaging System (Bio‐Rad, ChemiDoc MP, USA). The resulting densitometry values were normalized to the intensity levels of glyceraldehyde 3‐phosphate dehydrogenase (GAPDH), and quantification was performed using ImageJ.

### RNA Isolation and RNA Sequencing

Total RNA was meticulously extracted from mouse brain tissues employing the RNA isolater total RNA extraction reagent, a product sourced from Vazyme (Catalog No. R401‐01). Subsequently, these RNA samples were forwarded to Frasergen Genomic Medicine (Wuhan, China) for RNA sequencing (RNA‐seq). For optimizing sequencing results, an equivalent quantity of RNA extracted from three separate animal groups was pooled and used as the input material for RNA‐seq analysis.

The raw RNA‐seq reads were subjected to mapping onto the mouse genome via HISAT2 (version 2.2.1). Following this mapping step, quantification was conducted using FeatureCounts, and the resulting quantification dataset was subjected to further evaluation using R (version 4.2.2). Subsequently, the DESeq2 package was employed to discern DEGs. Genes were deemed differentially expressed if they exhibited an adjusted *p*‐value (*p* adj) of <0.05, coupled with an absolute value of log2FoldChange exceeding 0.58.

For a deeper understanding of the functional implications of these DEGs, KEGG pathway enrichment analysis and GO enrichment analysis were carried out using the clusterProfiler R package. In parallel, an additional dataset containing RNA‐seq data from AD (Alzheimer's disease) patients, derived from the GSE33000 dataset, was analyzed. The analysis of this dataset was conducted using the R package Limma (version 3.56.2).

### Brain Tissue Slices and Immunofluorescence Staining

The brains of the mice were subjected to a perfusion process as follows: they were first perfused with frozen PBS (pH 7.4) transcardially, followed by perfusion with a solution of 4% paraformaldehyde dissolved in PBS for tissue fixation. After this cardiac perfusion, the brains were carefully removed, placed in a solution of 4% paraformaldehyde, and stored in a light‐protected environment at 4 °C until further use. The brain tissue was allowed to undergo fixation for a minimum of 24 h before being transferred to a 30% sucrose solution for dehydration, a process that spanned 48 h. Once the tissue had sunk to the bottom of the solution, the brains were sectioned into 30‐µm‐thick coronal sections using a freezing microtome (Leica, CM1950). Sections of the brain that included an intact hippocampus were preserved in a freezing solution consisting of PBS, ethylene glycol, and glycerin in a ratio of 5:3:2 and stored at −20 °C.

Subsequently, the brain sections underwent a series of preparatory steps. They were washed with pre‐cooled PBS at room temperature for 5 min, repeating this process thrice. To prevent non‐specific binding, the sections were then blocked with a solution of 3% bovine serum albumin (BSA) prepared in PBS for a duration of 1 h at room temperature. Following the blocking step, primary antibodies, suitably diluted in BSA, were applied to the sections and allowed to incubate overnight at 4 °C. After the primary antibody incubation, the sections were washed with PBS at room temperature for 5 min, repeating this process three times.

Subsequent to the primary antibody incubation and washing steps, secondary antibodies labeled with Alexa Fluor Plus 488 and 594 were employed and allowed to incubate with the sections for 1 h at room temperature. Following this incubation, 4′,6‐diamidino‐2‐phenylindole (DAPI) was added to each section and allowed to incubate for 20 min at room temperature in darkness. The fluorescent images were then observed and captured using a confocal laser scanning microscope, specifically the LSM800 model from Zeiss, Germany. Image processing and analysis were performed utilizing the ZEN imaging software. Fluorescence signals were quantified using ImageJ v1.8.0.

### Determination of ROS in Brain Tissues

The brain slice samples were subjected to a specific staining procedure as follows: Dihydroethidium (DHE), obtained from Beyotime in China, was utilized for staining. The brain slices were incubated with DHE at a temperature of 37 °C for a duration of 30 min. Subsequently, the slices were thoroughly washed three times with PBS, with each washing step lasting for 5 min. To facilitate nuclear staining, the samples were further subjected to co‐staining with DAPI, also sourced from Beyotime in China.

The resulting stained samples were subjected to fluorescence intensity analysis using a confocal laser scanning microscope, specifically the Carl Zeiss LSM800 model. This analysis allowed for the visualization and quantification of the fluorescence signals in brain slices, providing valuable insights into the experimental observations.

### Real‐Time PCR

Total RNA was meticulously extracted from mice brain tissues utilizing the RNA isolater Total RNA Extraction Reagent, a product sourced from Vazyme (Catalog No. R401‐01). Subsequently, this isolated RNA was reverse‐transcribed into complementary DNA (cDNA) using the HiScript III RT SuperMix for qPCR (+gDNA wiper), a kit also obtained from Vazyme (Catalog No. R323). This process was conducted in strict accordance with the manufacturer's instructions.

Real‐time PCR was conducted employing the Taq Pro Universal SYBR qPCR Master Mix, procured from Vazyme (Catalog No. Q712). The PCR protocol consisted of an initial denaturation step at 95 °C for 30 s, followed by 40 amplification cycles, each cycle consisting of denaturation at 95 °C for 5 s, annealing at 60 °C for 30 s, and extension at 72 °C for 30 s. These PCR reactions were performed using the CFX96 Real‐Time PCR Detection system, a product of Bio‐Rad with version 2.2 of the software.

To determine relative expression changes, the 2^−ΔΔCt^ method was employed, wherein the quantity of the target gene expression was normalized to the expression of an endogenous control gene, GAPDH in this case. Specific primer sequences utilized for qPCR analysis are provided in Table S7 (Supporting Information).

### Long‐Term Potentiation (LTP) Recording

The procedure for brain tissue preparation and electrophysiological recordings was as in previous studies.^[^ ^59^, ^60^ ^]^ In detail, mice were anesthetized, and perfusion was carried out with a sucrose‐based solution through the left ventricle of the heart until the limbs turned white. The brain was swiftly removed and placed in a chilled sucrose‐based cutting solution. The brain was then sliced into 350‐µm‐thick sections using a Leica VT1000S vibratome. These sections were carefully prepared to include the ventral hippocampus. The prepared slices, now containing the ventral hippocampus, were transferred to an incubation chamber, and submerged in oxygenated ACSF (artificial cerebrospinal fluid) with the following composition: 126 mm NaCl, 3 mm KCl, 1.25 mm NaH_2_PO_4_, 2 mm MgSO_4_, 24 mm NaHCO_3_, 2 mm CaCl_2_, and 10 mm glucose. After an adequate equilibration period, the slices were transferred to an interface‐type recording chamber, where they were continuously perfused with ACSF maintained at a temperature of 32 °C.

Field potentials were recorded using a glass pipette filled with ACSF, and the resistance of the pipette was within the range of 2–3 MΩ. Field excitatory postsynaptic potentials (fEPSPs) were evoked by orthodromic stimulation of the Schaffer collateral/commissural fibers, using twisted 50‐µm nickel/chromium wires. Pulses of 0.1 ms duration were delivered at intervals of 20 s. Stimulus intensity was varied to establish a stimulus intensity–response relationship. Once a stable baseline was achieved, LTP was induced by delivering a single high‐frequency stimulation train consisting of 100 pulses at 100 Hz for 1 s at standard intensity. Changes in the slopes of fEPSPs were continuously recorded and analyzed as a function of time, allowing for the assessment of LTP induction and maintenance.

### Determination of ROS in SH‐SY5Y Cells

SH‐SY5Y cells were seeded into 24‐well plates and incubated overnight until reached ≈70% confluence. Cells in the control group were cultured in DMEM for 36 h, and in the model group were cultured with DMEM for 12 h and treated with 10 µm Aβ_1‐42_ for 24 h. In PQQ and EX527 treatment group, cells were pretreated with 5 µm PQQ or 5 µm PQQ with 2 µm EX527 for 12 h, and then treated with 10 µm Aβ_1‐42_ for 24 h. After modeling and drug intervention, the cells were incubated with 10 µm dihydroethidium (Beyotime) solution for 30 min at 37 °C, followed by rinsed with PBS thrice and further constrained with DAPI (Beyotime, China). Cells were visualized under a confocal scanning microscope (Zeiss LSM 800) and processed using the ZEN imaging software.

### Luciferase Activity Assay

The pGL3 luciferase reporter vector was used for constructing luciferase vectors. To identify the CREB‐binding site along the promoters of human *Cnr1* and *Cx3cr1* promoter, a series of *Cnr1* promoter and *Cx3cr1* promoters were synthesized and then inserted into the pGL3 vector, generating a series of luciferase plasmids containing *Cnr1* promoter (pGL‐Cnr1‐600, pGL‐Cnr1‐1400, and pGL‐Cnr1‐1800) and *Cx3cr1* promoter (pGL‐Cx3cr1‐1000, pGL‐Cx3cr1‐1500, and pGL‐Cx3cr1‐2000), respectively. Moreover, the deletion of CREB‐binding sites generated plasmids of the *Cnr1* promoter (pGL‐Cnr1‐1400Δ and pGL‐Cnr1‐1800Δ) and *Cx3cr1* promoter (pGL‐Cx3cr1‐1500Δ and pGL‐Cx3cr1‐2000Δ), respectively.

All plasmids were confirmed by sequencing. HEK293 cells were transfected with the plasmids and transfection efficiency was adjusted according to the manufacturer's protocol. A Renilla luciferase vector was transfected as an internal control. Luciferase activities were measured using the Dual‐Luciferase Reporter Assay System (Promega).

### Chromatin Immunoprecipitation (ChIP) Assay

Chromatin immunoprecipitation assays were performed using a kit according to the manufacturer's instructions (EZ‐ChIP, Millipore). After experiments, chromatin protein was cross‐linked to DNA by the addition of 1% formaldehyde. The cells were lysed in SDS sonication buffer after centrifugation, and the lysate was sonicated to break up the DNA into 100–500 bp fragments. IgG and CREB groups were added with 0.1 µL IgG and 10 µL antibody against CREB according to the manufacturer's protocol, respectively. All DNA samples were purified using the FastPure Cell/Tissue DNA Isolation Mini Kit (DC102, Vazyme) before qPCR analysis. The determination of the ChIP signal is calculated using this formula: % input = 1% × 2 ^ (CT_input_ – CT_sample_). The relative DNA content was normalized to the input DNA content for each sample.

### Statistical Analysis

All data are expressed as mean ± standard error of the mean using GraphPad Prism 8. Two‐group comparisons were analyzed using Student's *t*‐test. For comparisons of more than two groups, one‐way ANOVA was employed. Statistical significance is indicated by *p* < 0.05. Statistical analysis was carried out using GraphPad Prism 8 Software.

## Conflict of Interest

The authors declare no conflict of interest.

## Supporting information

Supporting Information

## Data Availability

Research data are not shared.
