# Supplementary material for: Deep Learning‐Driven Exploration of Pyrroloquinoline Quinone Neuroprotective Activity in Alzheimer's Disease
Source: Adv Sci (Weinh). 2024 Mar 7;11(18):2308970. doi: 10.1002/advs.202308970 (PMC11095145; doi:10.1002/advs.202308970)
Supplement: Supplementary file 1 — Supporting Information [file ADVS-11-2308970-s001.pdf]

ADVANCED SCIENCE
Open Access

Supporting Information

for *Adv. Sci.*, DOI 10.1002/advs.202308970

Deep Learning-Driven Exploration of Pyrroloquinoline Quinone Neuroprotective Activity in Alzheimer's Disease

Xinuo Li, Yuan Sun, Zheng Zhou, Jinran Li, Sai Liu, Long Chen, Yiting Shi, Min Wang, Zheying Zhu*, Guangji Wang* and Qiulun Lu**

Deep Learning-Driven Exploration of Pyrroloquinoline Quinone Neuroprotective Activity in Alzheimer's Disease

Xinuo Li[#], Yuan Sun[#], Zheng Zhou[#], Jinran Li, Sai Liu, Long Chen, Yiting Shi, Min Wang, Zheying Zhu [*], Guangji Wang [*], Qiulun Lu [*]

X. L., Y. S. Jiangsu Provincial Key Laboratory of Drug Metabolism and Pharmacokinetics, State Key Laboratory of Natural Medicines, China Pharmaceutical University, Nanjing 211166, China.
Z. Z. Department of Computer Science, RWTH Aachen University, 52074 Aachen, Germany.
Jiangsu Provincial Key Laboratory of Drug Metabolism and Pharmacokinetics, State Key Laboratory of Natural Medicines, China Pharmaceutical University, Nanjing 211166, China. *J. L., S. L., L. C., Y. S.* Jiangsu Provincial Key Laboratory of Drug Metabolism and Pharmacokinetics, State Key Laboratory of Natural Medicines, China Pharmaceutical University, Nanjing 211166, China.
M. W. Affiliated Brain Hospital of Nanjing Medical University, Nanjing 210029, China.
Z. Z. School of Pharmacy, The University of Nottingham, Nottingham NG7 2RD, UK.
G. W. Jiangsu Provincial Key Laboratory of Drug Metabolism and Pharmacokinetics, State Key Laboratory of Natural Medicines, China Pharmaceutical University, Nanjing 211166, China.
Q. L. Jiangsu Provincial Key Laboratory of Drug Metabolism and Pharmacokinetics, State Key Laboratory of Natural Medicines, China Pharmaceutical University, Nanjing 211166, China.

E-mail: qiulunlu@cpu.edu.cn; guangjiwang@hotmail.com; zheying.zhu@nottingham.ac.uk; xinuo.li@cpu.edu.cn

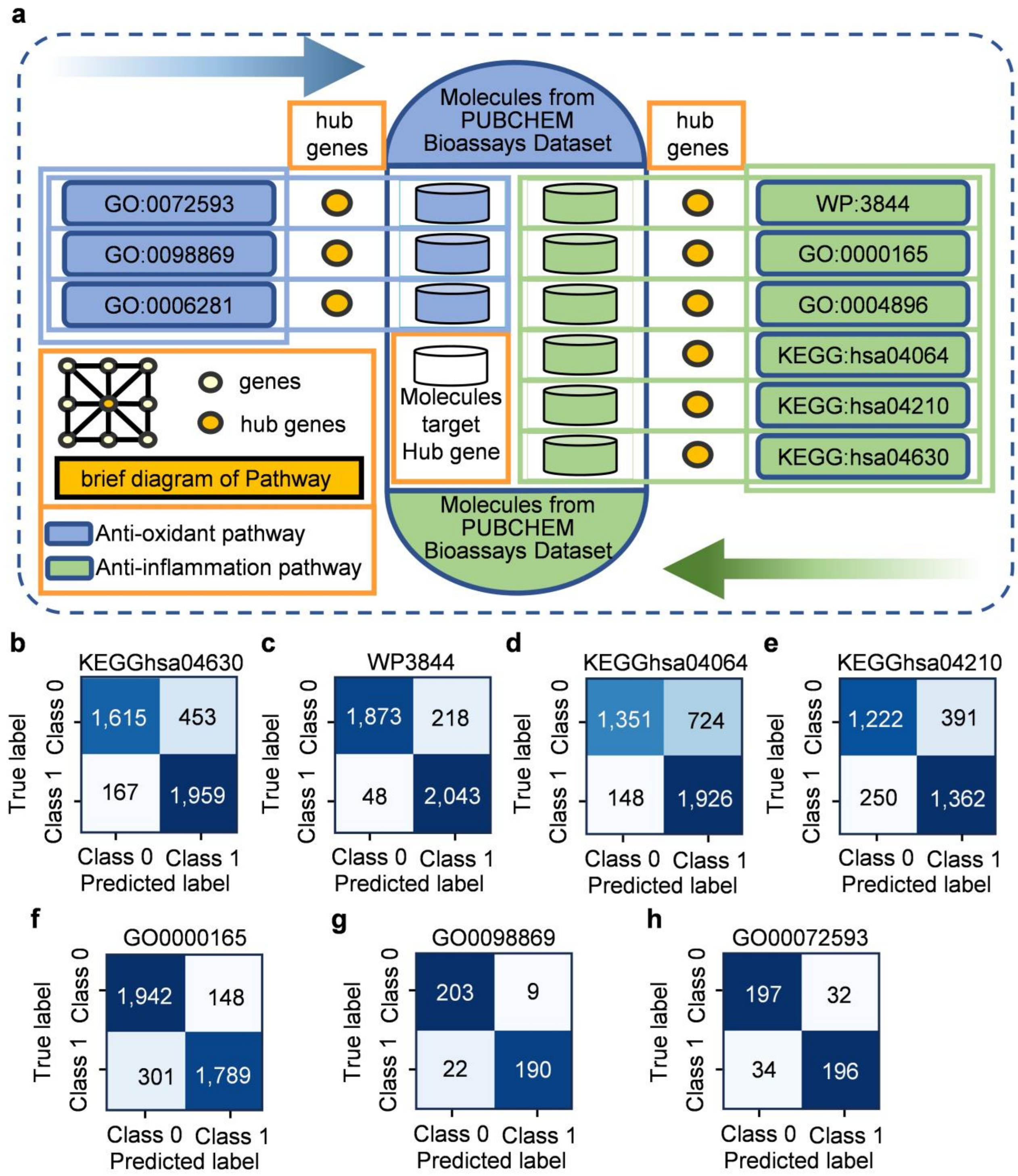

Supplementary Figure 1. Model establishment for anti-AD drug screening using deep learning. a. Overview of the preliminary data aggregation process utilized for the training and validation phases of the deep learning models. **b-f.** Confusion matrices for the five anti-inflammatory properties based on the test set results. **g and h.** Confusion matrices for the two antioxidant properties obtained using 5-fold cross-validation. For each channel, the matrix represents the validation results of the fold with the highest F1 score.

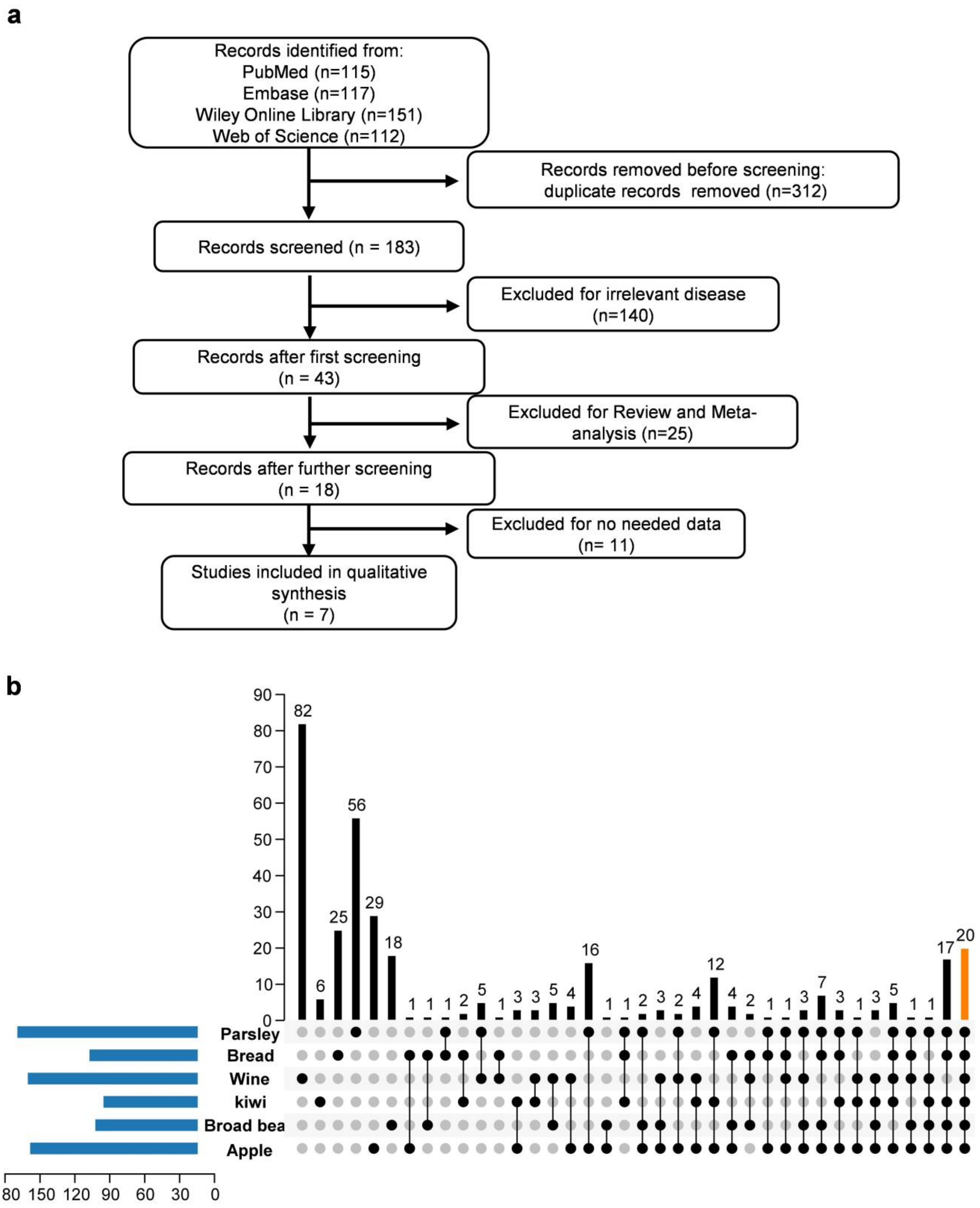

Supplementary Figure 2. Association between dietary patterns and AD risk. a. Meta-Analysis Guideline: An overview detailing the methodology for assessing the impact of the MIND dietary pattern on AD prevalence. **b.** Representative Food Components: A visual representation of the key components found in six emblematic foods associated with the MIND dietary pattern.

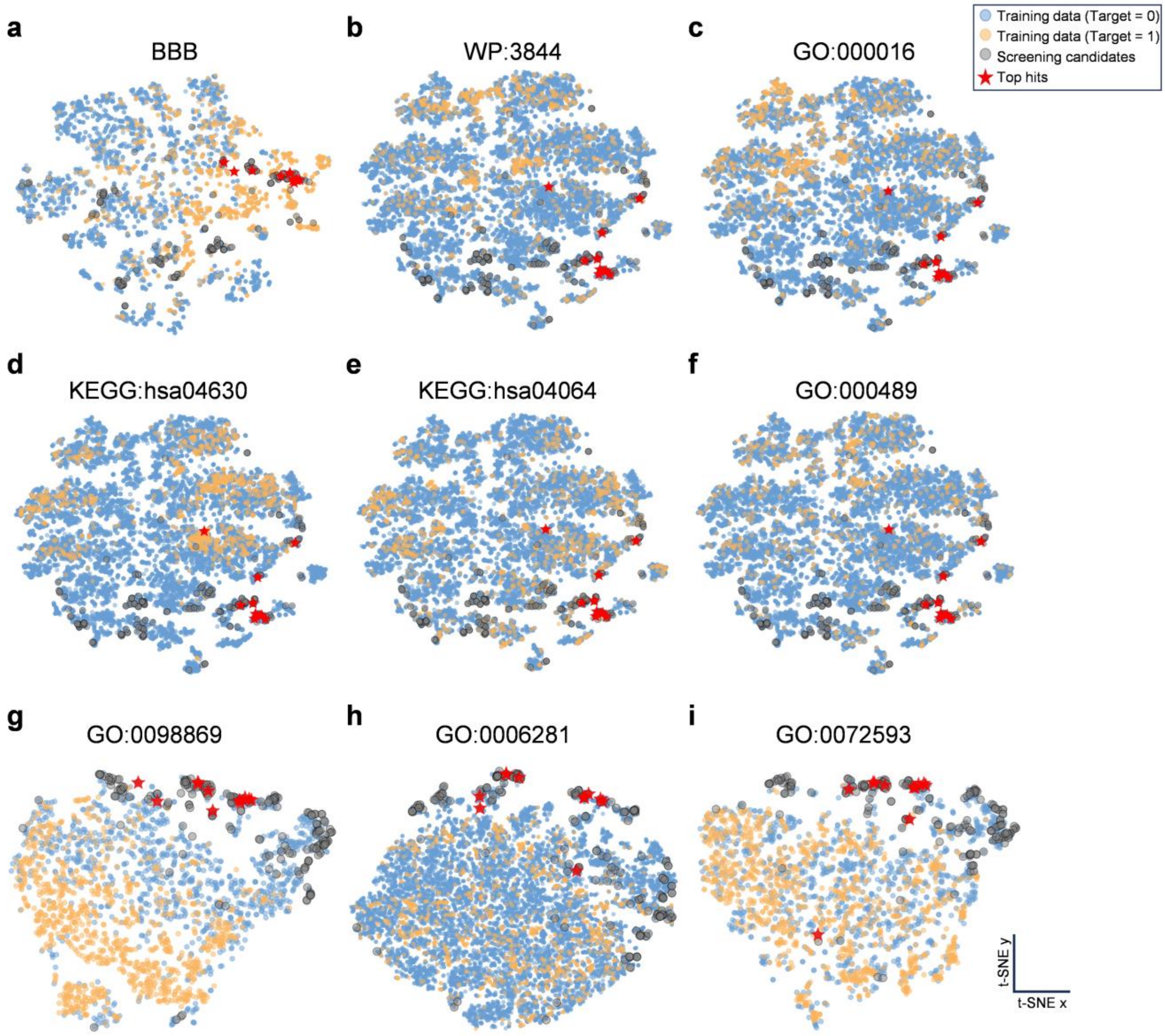

Supplementary Figure 3. All plots were generated using two-dimensional t-SNE visualizations with a perplexity of 30 and the maximum number of iterations set to 1000. The 12 highly scored non-toxic compounds ultimately selected are marked and showcased in each plot as “top hits”. **a-f.** (BBB and Anti-inflammatory): the compounds represented are derived from the original dataset, excluding any samples generated using data augmentation techniques such as SMOTE and ADASYN. **g-i.** (Anti-oxidative): the compounds displayed in these plots are selected from the original dataset; post systematic random downsampling procedure was applied to the majority class to achieve a balanced dataset.

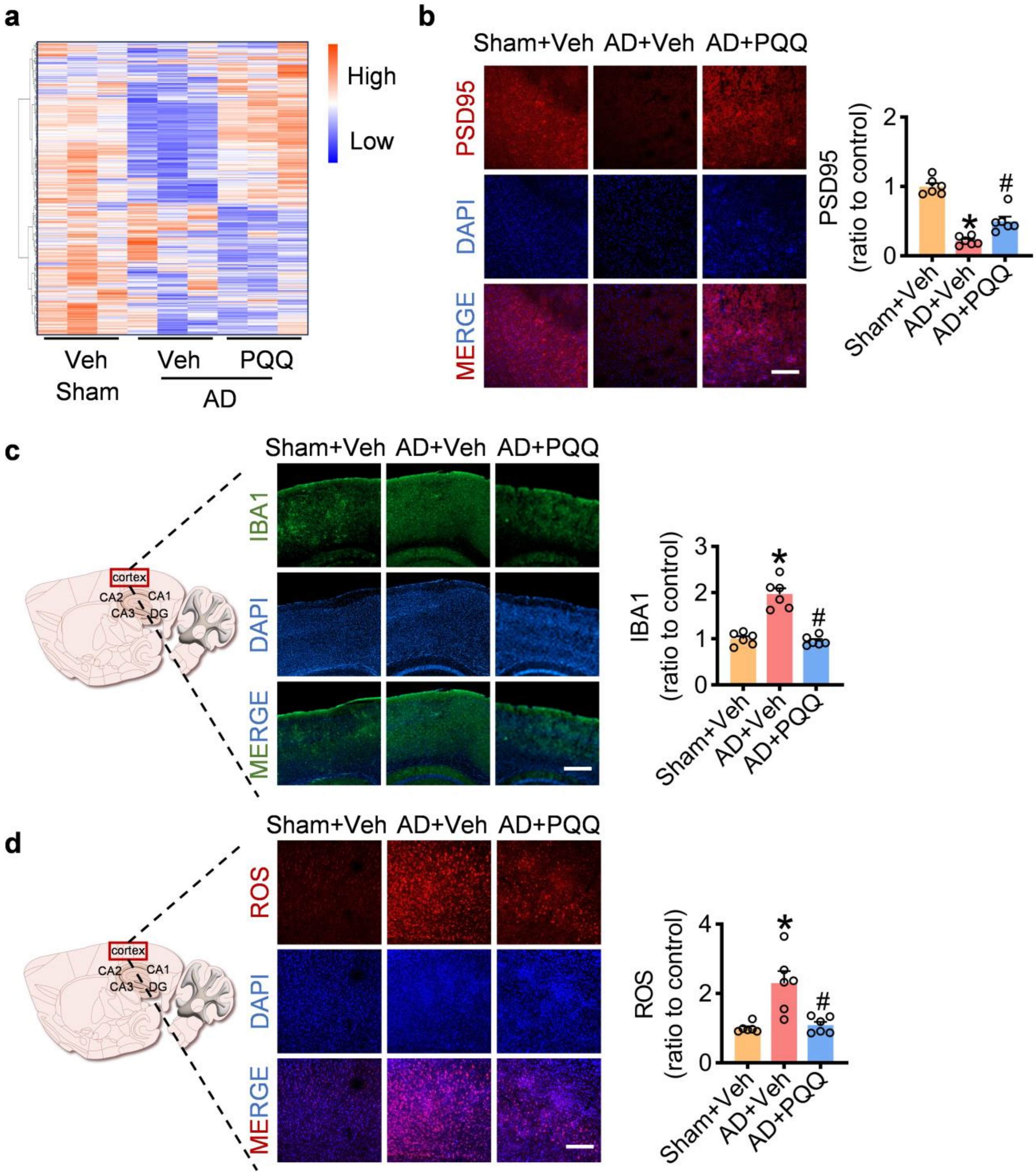

Supplementary Figure 4. PQQ's synaptic modulation, anti-inflammatory, and antioxidant mechanisms in AD mouse model. a. Heatmap representation of DEGs across control mice, and AD mice treated with either vehicle or PQQ. **b.** Representative fluorescence micrographs and statistical analysis of PSD95 in the cortex (scale bar = 200 μm, n = 6). **c** Representative fluorescence micrographs and statistical analysis of iBA1 in the cortex (Scale bar, 1000 μm, n = 6). **d.** Representative fluorescence micrographs and statistical analysis of DHE staining in the cortex (scale bar = 200 μm, n = 6). Data are shown as mean ± SEM. *P < 0.05 vs. control group; #P < 0.05 *vs.* AD group.

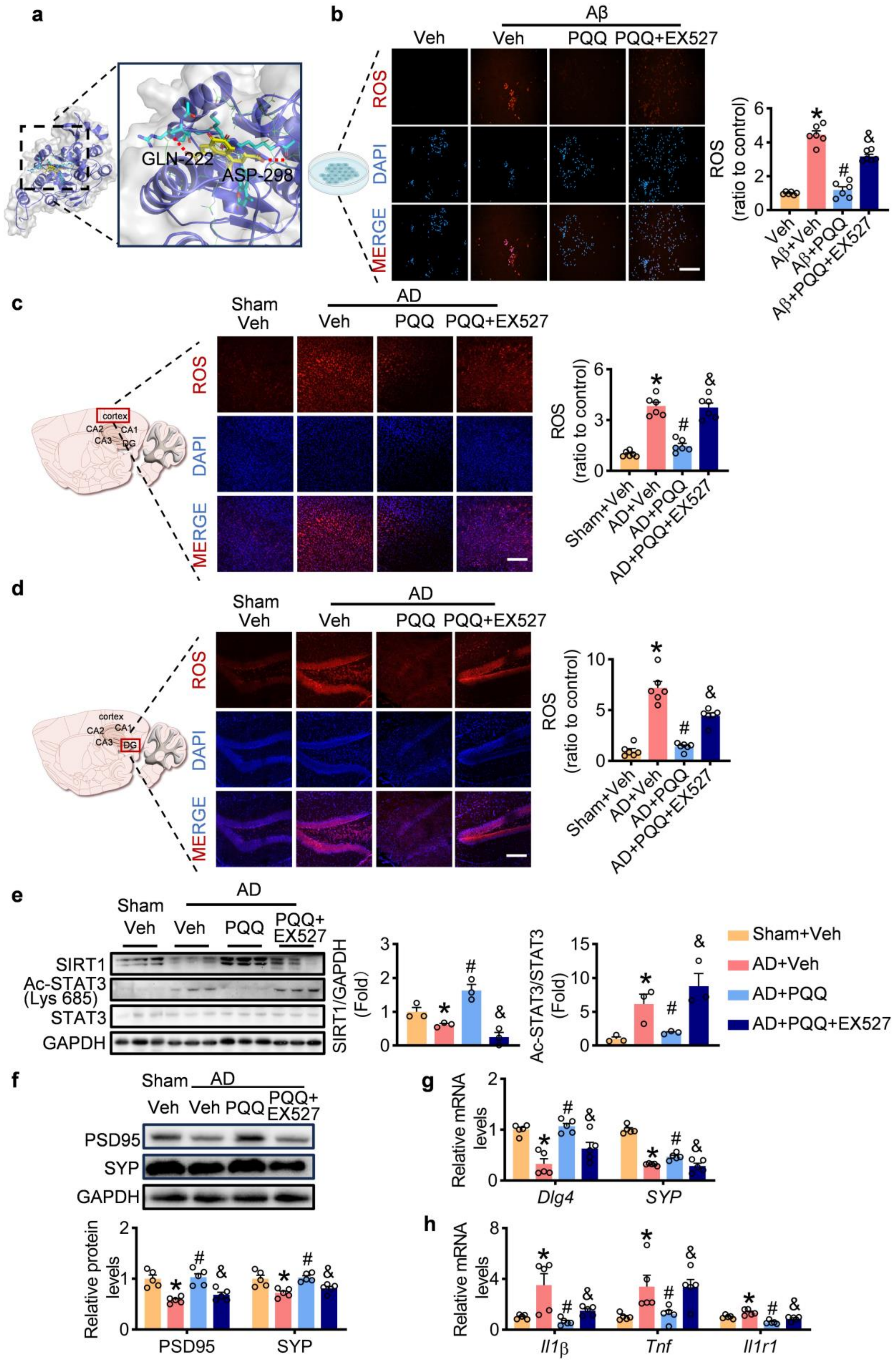

a
GLN-222
ASP-298
b
Aβ
Veh
Veh
PQQ
PQQ+EX527
ROS
DAPI
MERGE
ROS (ratio to control)
Veh
Aβ+Veh
Aβ+PQQ
Aβ+PQQ+EX527
c
Sham Veh
AD
Veh
PQQ
PQQ+EX527
cortex
CA2
CA1
CA3
DG
ROS
DAPI
MERGE
ROS (ratio to control)
Sham+Veh
AD+Veh
AD+PQQ
AD+PQQ+EX527
d
Sham Veh
AD
Veh
PQQ
PQQ+EX527
ROS
DAPI
MERGE
ROS (ratio to control)
e
Sham Veh
AD
Veh
PQQ
PQQ+ EX527
SIRT1
Ac-STAT3 (Lys 685)
STAT3
GAPDH
SIRT1/GAPDH (Fold)
Ac-STAT3/STAT3 (Fold)
Sham+Veh
AD+Veh
AD+PQQ
AD+PQQ+EX527
f
Sham
AD
Veh
Veh
PQQ
PQQ+ EX527
PSD95
SYP
GAPDH
Relative protein levels
PSD95
SYP
g
Relative mRNA levels
Dlg4
SYP
h
Relative mRNA levels
Il1β
Tnf
Il1r1

Supplementary Figure 5. Impact of SIRT1 inhibition on PQQ's neuroprotective mechanism. a. Illustration of PQQ's binding pocket within SIRT1. **b.** Representative fluorescence micrographs and statistical analysis of DHE staining in Aβ-injured SH-SH5Y cells (scale bar = 200 μm, n = 6). **c.** Representative fluorescence micrographs and statistical analysis of DHE staining in the cortex (scale bar = 200 μm, n = 6). **d.** Representative fluorescence micrographs and statistical analysis of DHE staining in the hippocampus (scale bar = 200 μm, n = 6). **e.** Representative SIRT1, STAT3, and AC-STAT3 bands and statistical analysis of SIRT1/ GAPDH and AC-STAT3/STAT3 expression levels (n = 3). **f.** Representative immunoblot images and subsequent statistical analysis of PSD95 and synaptophysin protein levels (n = 5). **g.** Quantitative assessment of relative mRNA expression levels of *PSD95* and synaptophysin (n = 5). **h.** Relative mRNA levels of *Il1β*, *Tnf*, and *Il1r1* (n = 5). Data are shown as mean ± SEM. $^{*}P < 0.05$ *vs.* control group; $^{\#}P < 0.05$ *vs.* AD group. $^{\&}P < 0.05$ *vs.* PQQ-treated group.

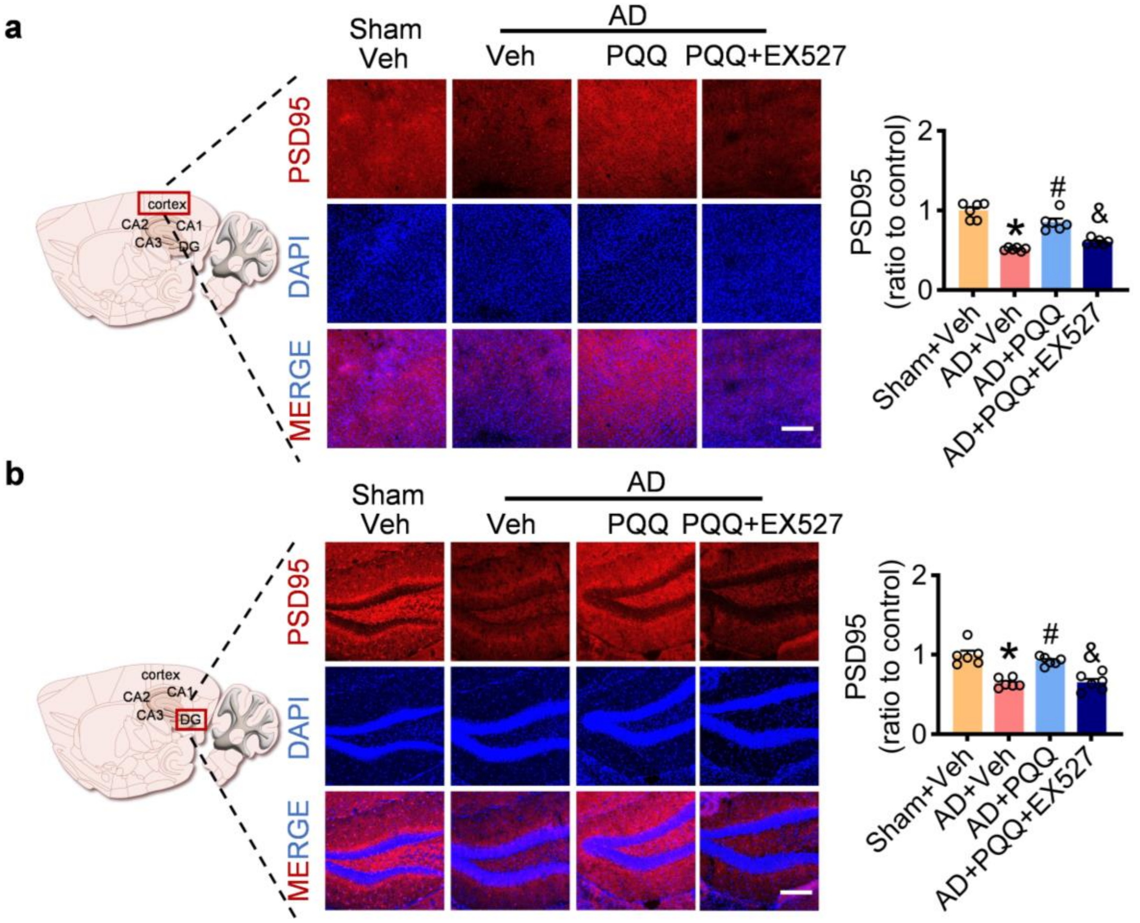

Supplementary Figure 6. Impact of SIRT1 inhibition on PQQ's synaptic function modulation. a. Representative fluorescence micrographs and statistical analysis of PSD95 in the cortex (scale bar = 200 μm, n = 6). **b.** Representative fluorescence micrographs and statistical analysis of PSD95 in the hippocampus (scale bar = 200 μm, n = 6). Data are shown as mean ± SEM. *P < 0.05 *vs.* control group; $^{\#}$P < 0.05 *vs.* AD group. $^{\&}$P < 0.05 *vs.* PQQ-treated group.

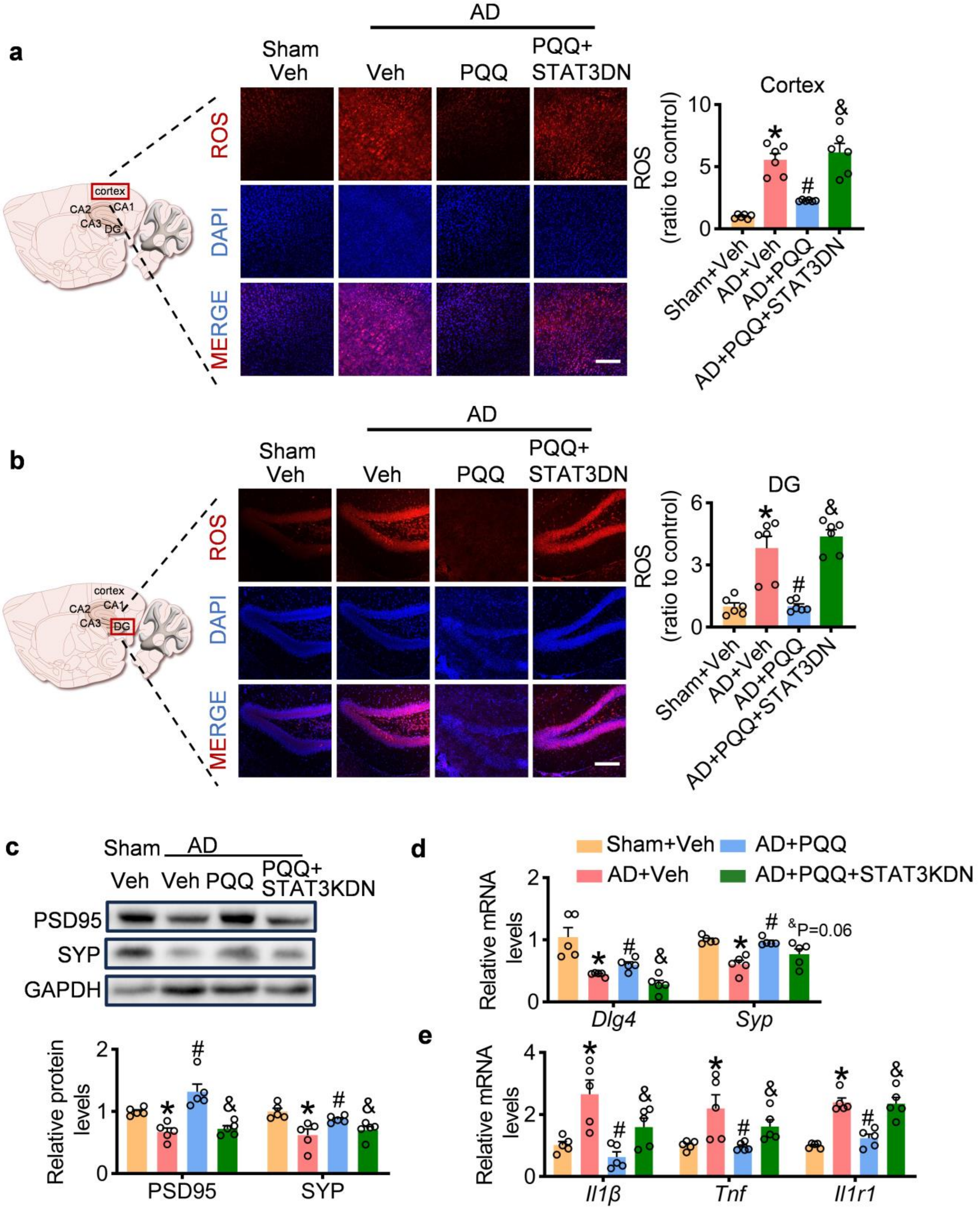

Supplementary Figure 7. Influence of STAT3 deacetylation on PQQ's neuroprotective efficacy. a. Fluorescence micrographs illustrating DHE staining in the cortical region, complemented by statistical analysis (scale bar = 200 μm, n = 6). **b.** Representative fluorescence micrographs and statistical analysis of DHE staining in the hippocampus (scale bar = 200 μm, n = 6). **c.** Representative immunoblot images and subsequent statistical analysis of PSD95 and synaptophysin protein levels (n = 5). **d.** Quantitative assessment of relative mRNA expression levels for *PSD95* and synaptophysin (n = 5). **e.** Relative mRNA levels of *Il1 β*, *Tnf*, and *Il1r1*(n = 5). Data are shown as mean ± SEM. *P < 0.05 vs. control group; $^{\#}$P < 0.05 *vs.* AD group. $^{\&}$P < 0.05 *vs.* PQQ-treated group.

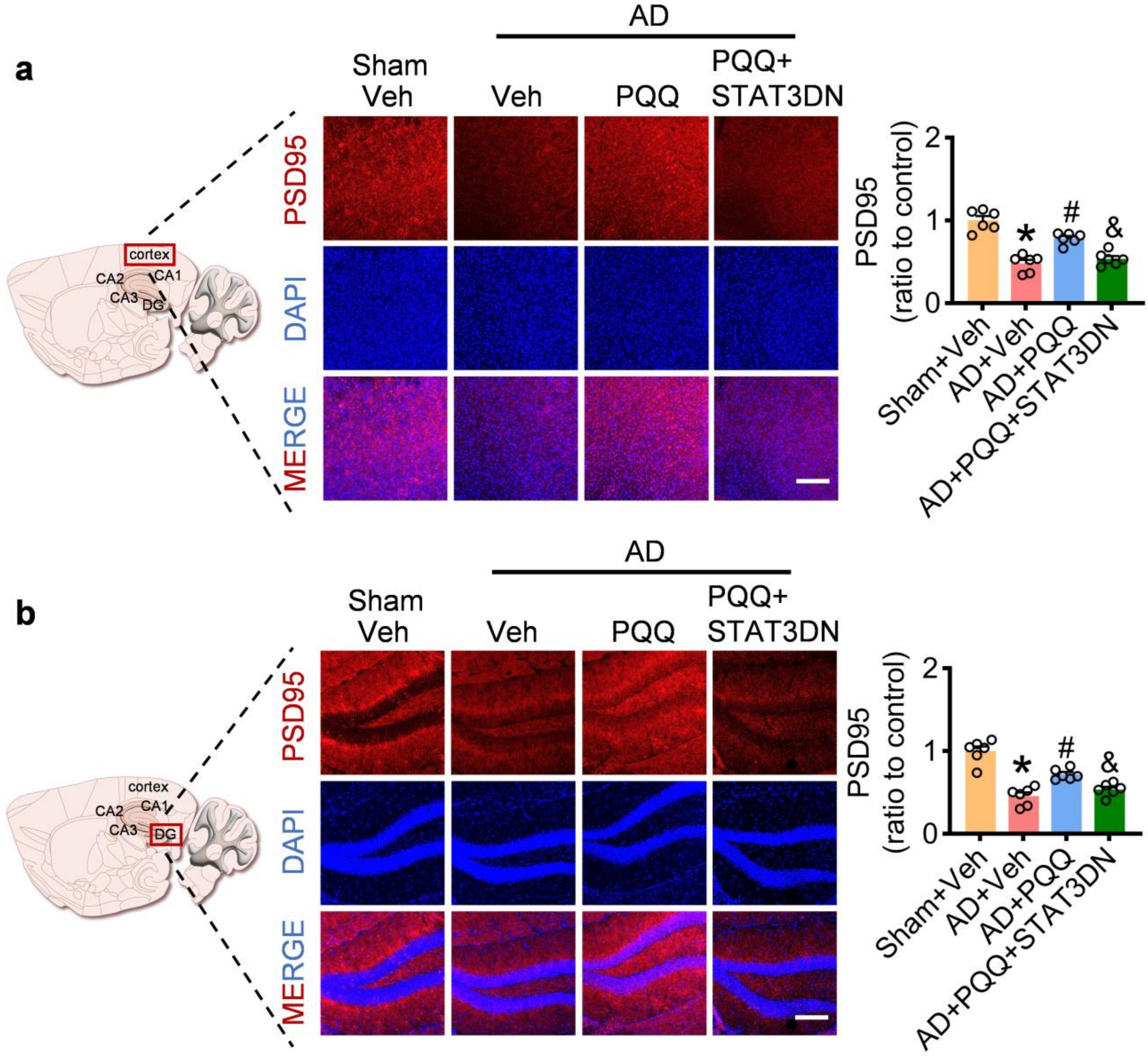

Supplementary Figure 8. Influence of STAT3 deacetylation on PQQ's synaptic function modulation. a. Representative fluorescence micrographs and statistical analysis of PSD95 in the cortex (scale bar = 200 μm, n = 6). **b.** Representative fluorescence micrographs and statistical analysis of PSD95 in the hippocampus (scale bar = 200 μm, n = 6). Data are shown as mean ± SEM. *P < 0.05 *vs.* control group; $^{\#}$P < 0.05 *vs.* AD group. $^{\&}$P < 0.05 *vs.* PQQ-treated group.

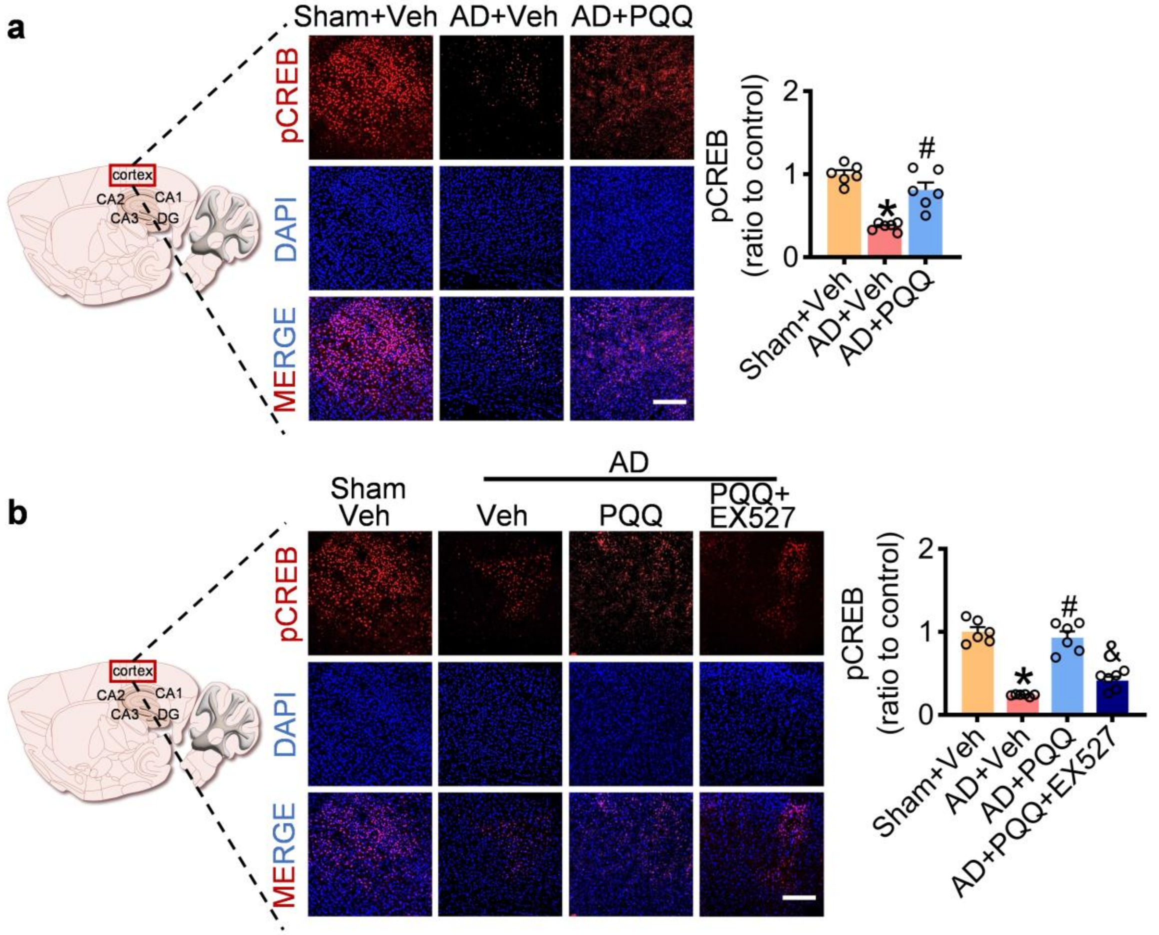

Supplementary Figure 9. PQQ modulates the CREB pathway in AD. a. **b.** Representative fluorescence micrographs and statistical analysis of p-CREB in the cortex (scale bar = 200 μm, n = 6). Data are shown as mean ± SEM (n = 6). *P < 0.05 *vs.* control group; #P < 0.05 vs.AD group. &P < 0.05 *vs.* PQQ-treated group.

Supplementary table 1

a. Configurations of MLP variants for BBB and anti-inflammatory properties

Name	Layers	epochs	focal_gamma	lin_first	lr	ps	sampler	use_early_stopping	use_scaler
BBB	[300, 100]	27	4.0313985	FALSE	0.001035774	0.100081474	SMOTE	TRUE	FALSE
WP:3844	[300, 200]	27	1.588732676	TRUE	0.002792903	0.101698207	SMOTE	TRUE	TRUE
GO:0000165	[350, 250]	26	1.858491874	TRUE	0.000412383	0.151492634	ADASYN	TRUE	TRUE
GO:0004896	[400, 300]	19	4.113254238	TRUE	0.003991385	0.103167061	SMOTE	TRUE	TRUE
KEGG:hsa04064	[400, 300]	22	4.148699864	FALSE	0.001365032	0.130046033	SMOTE	FALSE	FALSE
KEGG:hsa04210	[100, 200, 100]	30	2.385846152	TRUE	0.001628904	0.101429106	SMOTE	TRUE	TRUE
KEGG:hsa04630	[100, 200, 100]	29	4.723466049	TRUE	0.003085374	0.10713463	SMOTE	TRUE	TRUE

b. Configurations of GCN for antioxidant properties

Name	Graph Convolution Layers	Dropout	Predictor Hidden Features	Predictor Dropout	Activation	Residual	Batch Norm	Number Atom Features	Self Loop
GO:0006281	[64, 64]	0	128	0.5	None	TRUE	TRUE	30	TRUE
GO:0098869	[64, 64]	0	128	0.5	None	TRUE	TRUE	30	TRUE
GO:0072593	[121, 94, 59]	0.00984	202	0.06472	None	TRUE	TRUE	30	TRUE

Supplementary table 2

a. Binary Label Distribution of compounds across pathways and BBB permeability

Pathways	0	1
WP:3844	10449 (90.67%)	1075 (9.33%)
GO:0000165	10336 (89.69%)	1188 (10.31%)
GO:0004896	10771 (93.47%)	753 (6.53%)
KEGG:hsa04064	10372 (90%)	1152 (10%)
KEGG:hsa04210	8061 (69.95%)	3463 (30.05%)
KEGG:hsa04630	10455 (90.72%)	1069 (9.28%)
GO:0098869	10465 (90.81%)	1059 (9.19%)
GO:0072593	10377 (90.05%)	1147 (9.95%)
GO:0006281	10186 (88.39%)	1338 (11.61%)
BBB	3114 (71.67%)	1231 (28.33%)

b. The performance of the BBB and anti-inflammatory properties.

Pathways	Accuracy	Precision	Recall	F1 Score	ROC AUC
BBB	0.839486356	0.843902439	0.833065811	0.838449111	0.923588549
GO0000165	0.852169766	0.812189055	0.92144873	0.863375937	0.920855654
GO0004896	0.899512648	0.863905325	0.948491879	0.904224729	0.961378281
KEGGhsa04064	0.789828874	0.726792453	0.928640309	0.815410669	0.894429366
KEGGhsa04210	0.80124031	0.776953793	0.844913151	0.809509658	0.874845394
KEGGhsa04630	0.93639407	0.903582486	0.977044476	0.938878676	0.982152108
WP3844	0.892583732	0.923593185	0.855980861	0.888502607	0.964345253

c. The performance of the antioxidant properties.

Pathways	Accuracy	Precision	Recall	F1 Score	ROC AUC
GO0006281	0.86±0.04	0.84±0.04	0.89±0.08	0.86±0.05	0.94±0.03
GO0098869	0.88±0.05	0.87±0.08	0.91±0.03	0.89±0.04	0.96±0.02
GO0072593	0.8±0.04	0.8±0.04	0.8±0.08	0.8±0.05	0.88±0.04

Supplementary table 3 The 208 compounds from MIND

cid	ExactMolWt	MolLogP	TPSA	NumHDonors	NumHAcceptors	NumRotatableBonds	FractionCSP3	NumAromaticRings	MaxPartialCharge	MinPartialCharge	NumNitrogen	NumOxygen
19	154.0266	0.796	77.76	3	3	1	0	1	0.339119	-0.50424	0	4
127	152.0473	1.0193	57.53	2	2	2	0.125	1	0.307315	-0.50797	0	3
177	44.02621	0.2052	17.07	0	1	0	0.5	0	0.116405	-0.30372	0	1
247	117.079	-1.5575	40.13	0	2	2	0.8	0	0.118312	-0.54412	1	2
264	88.05243	0.8711	37.3	1	1	2	0.75	0	0.302829	-0.48123	0	2
273	102.1157	0.0741	52.04	2	2	4	1	0	-0.00773	-0.33047	2	0
284	46.00548	-0.2992	37.3	1	1	0	0	0	0.290129	-0.48347	0	2
305	104.107	-0.3151	20.23	1	1	2	1	0	0.101493	-0.39052	1	1
338	138.0317	1.0904	57.53	2	2	1	0	1	0.338969	-0.50705	0	3
370	170.0215	0.5016	97.99	4	4	1	0	1	0.335462	-0.50414	0	5
379	144.115	2.4315	37.3	1	1	6	0.875	0	0.30284	-0.48123	0	2
674	45.05785	-0.1644	12.03	1	1	0	1	0	-0.01675	-0.32277	1	0
702	46.04186	-0.0014	20.23	1	1	0	1	0	0.040221	-0.39666	0	1
753	92.04734	-1.6681	60.69	3	3	2	1	0	0.100047	-0.39359	0	3
849	129.079	0.2131	49.33	2	2	1	0.833333	0	0.320191	-0.48008	1	2
892	180.0634	-3.8346	121.38	6	6	0	1	0	0.110987	-0.3875	0	6
938	123.032	0.7798	50.19	1	2	1	0	1	0.336759	-0.4776	1	2
971	89.99531	-0.8444	74.6	2	2	0	0	0	0.414359	-0.47281	0	4
985	256.2402	5.5523	37.3	1	1	14	0.9375	0	0.30284	-0.48123	0	2
1024	330.0124	0.5503	174.72	4	6	3	0	2	0.354034	-0.47762	2	8
1031	60.05751	0.3887	20.23	1	1	1	1	0	0.042789	-0.39639	0	1
1045	88.10005	-0.316	52.04	2	2	3	1	0	-0.00768	-0.33047	2	0
1054	169.0739	0.08022	73.58	3	4	2	0.375	1	0.142142	-0.50566	1	3
1060	88.01604	-0.34	54.37	1	2	1	0.333333	0	0.371343	-0.47551	0	3
1130	265.1118	0.60774	75.91	2	5	4	0.416667	2	0.224996	-0.396	4	1
1146	59.0735	0.1778	3.24	0	1	0	1	0	-0.014	-0.31243	1	0
1183	152.0473	1.2133	46.53	1	3	2	0.125	1	0.160689	-0.50425	0	3
2969	172.1463	3.2117	37.3	1	1	8	0.9	0	0.30284	-0.48123	0	2
3893	200.1776	3.9919	37.3	1	1	10	0.916667	0	0.30284	-0.48123	0	2
5281	284.2715	6.3325	37.3	1	1	16	0.944444	0	0.30284	-0.48123	0	2
5570	137.0477	-1.1254	44.01	0	2	1	0.142857	1	0.177119	-0.54457	1	2
5780	182.079	-3.5854	121.38	6	6	5	1	0	0.110579	-0.39358	0	6
5793	180.0634	-3.2214	110.38	5	6	1	1	0	0.18357	-0.39357	0	6
5870	270.162	3.8174	37.3	1	2	0	0.611111	1	0.138556	-0.50796	0	2

5961	146.0691	-1.3362	106.41	3	3	4	0.6	0	0.319983	-0.48009	2	3
5991	296.1776	3.6126	40.46	2	2	0	0.6	1	0.130336	-0.50796	0	2
5997	386.3549	7.3887	20.23	1	1	5	0.925926	0	0.05774	-0.3928	0	1
6036	180.0634	-3.2214	110.38	5	6	1	1	0	0.18357	-0.39357	0	6
6047	197.0688	0.0522	103.78	4	4	3	0.222222	1	0.320316	-0.50426	1	4
6054	122.0732	1.2214	20.23	1	1	2	0.25	1	0.047094	-0.39603	0	1
6072	436.1369	-0.2024	177.14	7	10	7	0.380952	2	0.228764	-0.50797	0	10
6115	93.05785	1.2688	26.02	1	1	0	0	1	0.031348	-0.39873	1	0
6134	342.1162	-5.3972	189.53	8	11	4	1	0	0.186732	-0.39357	0	11
6202	336.0578	-1.96646	75.91	2	5	4	0.416667	2	0.224996	-1	4	1
6255	342.1162	-5.3972	189.53	8	11	4	1	0	0.186732	-0.39357	0	11
6306	131.0946	0.4444	63.32	2	2	3	0.833333	0	0.320247	-0.48008	1	2
6322	174.1117	-1.5481	127.72	4	3	5	0.666667	0	0.319969	-0.48009	4	2
6341	45.05785	-0.035	26.02	1	1	0	1	0	-0.01058	-0.33075	1	0
6405	88.08882	1.1673	20.23	1	1	1	1	0	0.05887	-0.39048	0	1
6560	74.07316	0.6347	20.23	1	1	1	1	0	0.045349	-0.39611	0	1
7504	107.0735	1.1453	26.02	1	1	1	0.142857	1	0.017761	-0.3265	1	0
8021	73.08915	0.6158	12.03	1	1	2	1	0	-0.00775	-0.31728	1	0
8060	87.1048	1.1353	26.02	1	1	3	1	0	-0.00773	-0.33047	1	0
8083	87.06841	-0.3938	21.26	1	2	0	1	0	0.059108	-0.37881	1	1
8094	130.0994	2.0414	37.3	1	1	5	0.857143	0	0.30284	-0.48123	0	2
8158	158.1307	2.8216	37.3	1	1	7	0.888889	0	0.30284	-0.48123	0	2
8471	101.1204	1.3481	3.24	0	1	3	1	0	-0.00474	-0.30416	1	0
8655	182.0579	1.2219	55.76	1	4	3	0.222222	1	0.20028	-0.5017	0	4
8742	174.0528	-1.5162	97.99	4	4	1	0.571429	0	0.33093	-0.47788	0	5
8768	138.0317	0.9103	57.53	2	3	1	0	1	0.157608	-0.50426	0	3
8857	88.05243	0.5694	26.3	0	2	1	0.75	0	0.302113	-0.46617	0	2
8892	116.0837	1.6513	37.3	1	1	4	0.833333	0	0.30284	-0.48123	0	2
9064	290.079	1.5461	110.38	5	6	1	0.2	2	0.157305	-0.50768	0	6
10393	138.0681	0.927	40.46	2	2	2	0.25	1	0.115095	-0.50797	0	2
10467	312.3028	7.1127	37.3	1	1	18	0.95	0	0.30284	-0.48123	0	2

10690	196.0583	-3.4931	138.45	6	6	5	0.833333	0	0.334877	-0.47925	0	7
10742	198.0528	1.1076	75.99	2	4	3	0.222222	1	0.335463	-0.5017	0	5
11005	228.2089	4.7721	37.3	1	1	12	0.928571	0	0.30284	-0.48123	0	2
11503	135.1048	1.4485	12.03	1	1	3	0.333333	1	-0.00114	-0.31942	1	0
12025	85.05276	-0.1036	29.1	1	1	0	0.75	0	0.219562	-0.35609	1	1
13250	198.0528	0.9801	86.99	3	5	2	0.222222	1	0.337891	-0.50414	0	5
13849	242.2246	5.1622	37.3	1	1	13	0.933333	0	0.30284	-0.48123	0	2
14985	430.3811	8.84026	29.46	1	2	12	0.793103	1	0.126525	-0.50738	0	2
31260	88.08882	1.0248	20.23	1	1	2	1	0	0.043296	-0.39637	0	1
64689	180.0634	-3.2214	110.38	5	6	1	1	0	0.18357	-0.39357	0	6
65084	306.074	1.2517	130.61	6	7	1	0.2	2	0.199777	-0.50768	0	7
68245	338.0193	-0.3815	132.68	6	6	1	0	3	0.401735	-1	0	7
72276	290.079	1.5461	110.38	5	6	1	0.2	2	0.157305	-0.50768	0	6
72277	306.074	1.2517	130.61	6	7	1	0.2	2	0.199777	-0.50768	0	7
72281	302.079	2.5185	96.22	3	6	2	0.1875	2	0.173909	-0.50768	0	6
73399	358.1416	3.1902	77.38	2	6	4	0.4	2	0.160347	-0.50425	0	6
79803	69.05785	0.851	12.36	0	1	0	0.75	0	0.03884	-0.29752	1	0
82755	154.063	0.6326	60.69	3	3	2	0.25	1	0.157164	-0.50426	0	3
88000	305.0859	-4.1759	191.12	7	9	3	0.6	1	0.326957	-0.45238	3	8
92094	402.3498	8.22342	29.46	1	2	12	0.777778	1	0.125993	-0.50794	0	2
107905	442.09	2.5276	177.14	7	10	3	0.136364	3	0.338339	-0.50768	0	10
119205	358.1416	2.6894	85.22	2	6	6	0.35	2	0.309204	-0.50425	0	6
119258	450.1162	0.0381	186.37	7	11	3	0.380952	2	0.202866	-0.50768	0	11
122738	578.1424	2.995	220.76	10	12	3	0.2	4	0.157305	-0.50768	0	12
128861	287.055	2.9089	112.45	5	5	1	0	3	0.401596	-0.50745	0	6
147299	578.1424	2.995	220.76	10	12	3	0.2	4	0.157305	-0.50768	0	12
152059	452.3654	9.2375	34.14	0	2	15	0.677419	1	0.189655	-0.28895	0	2
157643	466.1111	-0.2563	206.6	8	12	3	0.380952	2	0.202866	-0.50768	0	12
160521	360.1573	2.02	99.38	4	6	5	0.4	2	0.160272	-0.50425	0	6
160544	286.0841	3.0954	65.11	0	5	3	0.3125	3	0.335697	-0.48921	0	5
169853	866.2058	4.4439	331.14	15	18	5	0.2	6	0.157305	-0.50768	0	18
171548	244.0882	0.7968	78.43	3	3	5	0.8	0	0.314936	-0.48123	2	3

181681	388.1522	3.1988	86.61	2	7	5	0.428571	2	0.200254	-0.50425	0	7
222284	414.3862	8.0248	20.23	1	1	6	0.931034	0	0.05774	-0.3928	0	1
237332	126.0317	0.5844	50.44	1	3	2	0.166667	1	0.18482	-0.45584	0	3
332427	360.1573	2.6537	88.38	3	6	6	0.4	2	0.160347	-0.50425	0	6
439195	150.0528	-2.5823	90.15	4	5	0	1	0	0.183115	-0.3879	0	5
439215	194.0427	-3.1291	127.45	5	6	1	0.833333	0	0.335291	-0.47924	0	7
439230	148.0736	-0.4055	77.76	3	3	4	0.833333	0	0.305758	-0.48115	0	4
439246	272.0685	2.5099	86.99	3	5	1	0.133333	2	0.173909	-0.50797	0	5
440832	271.0601	3.2033	92.22	4	4	1	0	3	0.401456	-0.50797	0	5
441773	301.0707	3.2119	101.45	4	5	2	0.0625	3	0.401596	-0.50745	0	6
443023	418.1628	3.2074	95.84	2	8	6	0.454545	2	0.200254	-0.5017	0	8
443650	465.1028	0.0876	211.83	9	11	4	0.285714	3	0.40205	-0.50745	0	12
443651	479.1184	0.3906	200.83	8	11	5	0.318182	3	0.40205	-0.50745	0	12
445354	286.2297	5.5103	20.23	1	1	5	0.5	0	0.06175	-0.39229	0	1
445580	328.2402	6.5489	37.3	1	1	14	0.409091	0	0.303155	-0.48122	0	2
445639	282.2559	6.1085	37.3	1	1	15	0.833333	0	0.30284	-0.48123	0	2
446284	302.2246	5.9927	37.3	1	1	13	0.45	0	0.302851	-0.48123	0	2
452110	733.5622	10.6118	111.19	0	8	38	0.95	0	0.305766	-0.75612	1	8
484757	454.1416	5.0786	121.38	6	6	2	0.142857	4	0.122887	-0.50797	0	6
493570	376.1383	-1.72356	161.56	5	9	5	0.411765	1	0.349448	-0.39358	4	6
637098	454.1416	5.6506	110.38	5	6	4	0.071429	4	0.134737	-0.50797	0	6
637517	282.2559	6.1085	37.3	1	1	15	0.833333	0	0.30284	-0.48123	0	2
637540	164.0473	1.49	57.53	2	2	2	0	1	0.327824	-0.50733	0	3
637542	164.0473	1.49	57.53	2	2	2	0	1	0.327821	-0.50797	0	3
643733	258.0892	2.6181	56.51	0	4	4	0.2	1	0.330102	-0.46585	0	4
656516	457.1584	-3.10802	202.32	7	12	7	0.65	1	0.188266	-0.39357	1	11
667639	244.0736	2.6794	80.92	4	4	2	0	2	0.15749	-0.5078	0	4
689043	180.0423	1.1956	77.76	3	3	2	0	1	0.327821	-0.50426	0	4

1794427	354.0951	-0.6459	164.75	6	8	4	0.375	1	0.335432	-0.50426	0	9
5276454	442.09	2.5276	177.14	7	10	3	0.136364	3	0.338339	-0.50768	0	10
5280343	302.0427	1.988	131.36	5	7	1	0	3	0.238289	-0.50768	0	7
5280417	330.074	2.594	109.36	3	7	3	0.117647	3	0.2385	-0.50689	0	7
5280445	286.0477	2.2824	111.13	4	6	1	0	3	0.196579	-0.50768	0	6
5280450	280.2402	5.8845	37.3	1	1	14	0.722222	0	0.30284	-0.48123	0	2
5280459	448.1006	0.4887	190.28	7	11	3	0.285714	3	0.238633	-0.50768	0	11
5280489	536.4382	12.6058	0	0	0	10	0.45	0	-0.0104	-0.06957	0	0
5280795	384.3392	7.619	20.23	1	1	6	0.777778	0	0.058338	-0.39277	0	1
5280804	464.0955	-0.5389	210.51	8	12	4	0.285714	3	0.238633	-0.50768	0	12
5280805	610.1534	-1.6871	269.43	10	16	6	0.444444	3	0.238633	-0.50768	0	16
5280845	466.3447	8.3688	46.67	0	3	14	0.677419	1	0.198724	-0.34498	0	3
5280863	286.0477	2.2824	111.13	4	6	1	0	3	0.238289	-0.50797	0	6
5280934	278.2246	5.6605	37.3	1	1	13	0.611111	0	0.30284	-0.48123	0	2
5281127	282.2559	6.1085	37.3	1	1	15	0.833333	0	0.30284	-0.48123	0	2
5281222	272.0685	2.4051	97.99	4	5	3	0	2	0.189075	-0.50782	0	5
5281235	552.4331	11.5766	20.23	1	1	10	0.45	0	0.058527	-0.39276	0	1
5281243	568.428	10.4033	40.46	2	2	10	0.45	0	0.07286	-0.39276	0	2
5281618	284.0685	2.8798	79.9	2	5	2	0.0625	3	0.192821	-0.50782	0	5
5281643	464.0955	-0.5389	210.51	8	12	4	0.285714	3	0.238633	-0.50768	0	12
5281672	318.0376	1.6936	151.59	6	8	1	0	3	0.238289	-0.50768	0	8
5281712	406.1264	0.1525	160.07	7	9	5	0.3	2	0.228725	-0.5078	0	9

5281728	454.1416	5.6506	110.38	5	6	4	0.071429	4	0.134763	-0.50797	0	6
5281766	338.1002	-0.3515	144.52	5	7	4	0.375	1	0.335388	-0.50797	0	8
5282073	254.0579	2.8712	70.67	2	4	1	0	3	0.192821	-0.50797	0	4
5282102	448.1006	-0.2445	190.28	7	11	4	0.285714	3	0.238633	-0.50797	0	11
5282767	310.2872	6.8887	37.3	1	1	17	0.85	0	0.30284	-0.48123	0	2
5284607	450.3498	9.1576	34.14	0	2	14	0.612903	1	0.189972	-0.28895	0	2
5287939	648.5845	13.7531	26.3	0	2	20	0.844444	0	0.305565	-0.46202	0	2
5311498	1354.567	3.40921	488.71	9	20	26	0.619048	2	3	-0.7561	14	14
5316673	432.1056	0.7831	170.05	6	10	3	0.285714	3	0.238633	-0.50797	0	10
5318645	478.1111	-0.2359	199.51	7	12	5	0.318182	3	0.238633	-0.50768	0	12
5318767	594.1585	-1.3927	249.2	9	15	6	0.444444	3	0.238633	-0.50797	0	15
5321278	434.0849	0.1002	190.28	7	11	4	0.25	3	0.238633	-0.50768	0	11
5490064	434.0849	0.1002	190.28	7	11	4	0.25	3	0.238633	-0.50768	0	11
5748352	892.5353	9.66962	126.84	0	8	20	0.545455	1	2	-0.87426	4	5
6419725	536.4382	12.4617	0	0	0	10	0.45	0	0.002851	-0.08497	0	0
6429333	146.0732	2.2888	17.07	0	1	2	0.1	1	0.149819	-0.29789	0	1
6436907	646.5689	13.5291	26.3	0	2	19	0.8	0	0.305565	-0.46202	0	2
6441280	338.1002	-0.3515	144.52	5	7	4	0.375	1	0.335432	-0.50797	0	8
6857447	416.3654	8.53184	29.46	1	2	12	0.785714	1	0.126259	-0.50766	0	2
10095180	448.1006	-0.2445	190.28	7	11	4	0.285714	3	0.238289	-0.50797	0	11
10372299	670.5689	13.8613	26.3	0	2	19	0.723404	0	0.305577	-0.46202	0	2
11249520	528.1035	-2.3024	189.83	7	11	6	0.347826	3	0.402051	-1	0	12

11250133	578.1424	2.995	220.76	10	12	3	0.2	4	0.157305	-0.50768	0	12
12303220	484.0772	-2.614	191.6	8	10	4	0.285714	3	0.401911	-1	0	11
13751990	866.2058	4.4439	331.14	15	18	5	0.2	6	0.157305	-0.50768	0	18
13831068	610.1323	2.4062	261.22	12	14	3	0.2	4	0.199777	-0.50768	0	14
13990893	578.1424	2.995	220.76	10	12	3	0.2	4	0.157305	-0.50768	0	12
14311152	498.0929	-2.311	180.6	7	10	5	0.318182	3	0.401911	-1	0	11
14353459	652.1639	-1.4674	275.5	9	17	7	0.448276	3	0.30222	-0.50689	0	17
15385440	507.1133	0.6584	217.9	8	12	5	0.304348	3	0.40205	-0.50745	0	13
15714477	491.1184	0.9528	197.67	7	11	5	0.304348	3	0.401911	-0.50745	0	12
16131430	906.2676	10.7216	220.76	10	12	7	0.107143	8	0.134763	-0.50797	0	12
21674154	625.1552	1.8303	226.21	7	13	7	0.258065	5	0.34708	-0.50426	0	14
44256932	1005.251	-1.592	419.57	14	24	15	0.422222	4	0.40205	-0.50797	0	26
44256961	521.129	0.9614	206.9	7	12	6	0.333333	3	0.40205	-0.50745	0	13
44257035	609.1603	2.1247	205.98	6	12	7	0.258065	5	0.34708	-0.50797	0	13
45109789	262.1181	-0.1362	127.45	5	5	4	1	0	0.46917	-0.39666	0	7
53477794	678.6315	14.7573	26.3	0	2	23	0.893617	0	0.305565	-0.46202	0	2
53477891	676.6158	14.5333	26.3	0	2	22	0.851064	0	0.305565	-0.46202	0	2
54682930	162.0317	1.4986	50.44	1	3	0	0	2	0.339446	-0.50691	0	3
57397583	740.2164	-2.892	308.12	11	19	8	0.545455	3	0.238633	-0.50797	0	19
72193652	505.1341	1.2558	186.67	6	11	6	0.333333	3	0.401911	-0.50745	0	12
72551457	326.0638	-0.1441	150.59	4	7	7	0.214286	1	0.347974	-0.50425	0	9
74977116	535.1446	1.2644	195.9	6	12	7	0.36	3	0.402051	-0.50745	0	13

75228679	1492.936	15.5225	242.61	1	17	74	0.753086	0	1	-0.75612	0	17
101422354	578.1636	-1.0983	228.97	8	14	6	0.444444	3	0.234534	-0.50797	0	14
101746085	568.1792	-1.7391	236.06	9	14	10	0.5	2	0.229175	-0.50797	0	14
102157736	636.169	-1.173	255.27	8	16	7	0.448276	3	0.30222	-0.50797	0	16
102470786	652.1639	-1.4674	275.5	9	17	7	0.448276	3	0.30222	-0.50689	0	17
117842555	617.1163	-2.4773	320.41	10	13	17	0.347826	1	0.347974	-0.50423	3	15
131752138	470.0883	-0.0771	183.21	6	10	7	0.3	2	0.445975	-0.5078	0	11
131752272	639.1708	2.6635	216.13	7	13	9	0.25	4	0.402051	-0.50797	0	14
131752297	769.2338	1.4032	265.82	9	16	11	0.368421	4	0.401911	-0.50797	0	17
135398658	441.1397	-0.0448	213.28	6	9	9	0.210526	3	0.325721	-0.48123	7	6
135413566	304.1019	-3.887	197.17	7	10	3	0.6	1	0.29657	-0.45233	4	7
156614133	544.1276	-6.6325	310.66	11	15	7	0.842105	0	0.335304	-0.48095	0	18
157009725	625.1552	2.3605	227.13	8	13	8	0.225806	4	0.40205	-0.50797	0	14
157009726	655.1657	2.3691	236.36	8	14	9	0.25	4	0.402051	-0.50745	0	15
157009736	784.2062	-2.1897	334.42	11	20	9	0.529412	3	0.505354	-0.50797	0	21
157009738	922.1844	-4.5075	479.23	15	19	28	0.424242	1	0.347974	-0.50346	6	21
157010024	782.2269	-2.3212	314.19	10	20	9	0.542857	3	0.30222	-0.50797	0	20

Supplementary table 4 The score of 208 compounds from MIND predicted by our dual-path model.

CID	BBB	WP:3844	GO:0000165	GO:0004896	KEGG:hsa04064	KEGG:hsa04210	KEGG:hsa04630	GO:0006281	GO:0072593	GO:0098869	score
439246	1	1	1	1	1	1	0	0	1	0	7
72281	1	1	1	1	1	1	0	0	1	0	7
21674154	1	0	0	1	1	1	0	1	0	0	5
73399	0	1	1	1	1	1	0	0	0	0	5
443023	1	0	1	1	1	1	0	0	0	0	5
6072	1	0	1	1	1	1	0	0	0	0	5
667639	1	0	0	1	1	1	0	1	0	0	5
5280805	1	0	1	1	1	1	0	0	0	0	5
5282073	1	0	0	1	1	1	0	0	1	0	5
5318645	1	0	1	1	1	1	0	0	0	0	5
5318767	1	0	1	1	1	1	0	0	0	0	5
1024	1	0	0	0	0	1	1	0	1	1	5
14353459	1	0	1	1	1	1	0	0	0	0	5
8768	1	0	1	0	1	1	0	0	1	0	5
44257035	1	0	0	1	1	1	0	1	0	0	5
57397583	1	0	1	1	1	1	0	0	0	0	5
102157736	1	0	1	1	1	1	0	0	0	0	5
102470786	1	0	1	1	1	1	0	0	0	0	5
101422354	1	0	1	1	1	1	0	0	0	0	5
101746085	1	0	1	1	1	1	0	0	0	0	5
72277	1	0	0	0	1	1	0	1	0	0	4
181681	0	0	1	1	1	1	0	0	0	0	4
117842555	1	0	0	0	1	1	0	0	1	0	4
131752297	1	0	0	1	1	1	0	0	0	0	4
440832	1	0	0	1	0	1	0	1	0	0	4
9064	1	0	0	0	1	1	0	1	0	0	4
439215	1	0	0	1	1	1	0	0	0	0	4
332427	1	0	0	1	1	1	0	0	0	0	4
160544	0	0	1	0	1	1	0	1	0	0	4
157009736	1	0	0	1	1	1	0	0	0	0	4
128861	1	0	0	0	1	1	0	1	0	0	4
119205	1	0	1	0	1	1	0	0	0	0	4
65084	1	0	0	0	1	1	0	1	0	0	4
82755	1	0	0	0	1	1	0	1	0	0	4
135398658	1	0	1	0	0	1	0	0	0	1	4
72276	1	0	0	0	1	1	0	1	0	0	4
443650	1	0	0	0	1	1	0	1	0	0	4
5280445	1	1	0	0	1	1	0	0	0	0	4
1794427	1	0	0	1	1	1	0	0	0	0	4
5281712	1	0	0	1	1	1	0	0	0	0	4
10095180	1	0	1	0	1	1	0	0	0	0	4
6429333	0	0	1	0	1	1	0	0	1	0	4
5490064	1	0	1	0	1	1	0	0	0	0	4
5321278	1	0	1	0	1	1	0	0	0	0	4
72551457	1	0	0	0	1	1	0	0	1	0	4
5316673	1	0	1	0	1	1	0	0	0	0	4
5282102	1	0	1	0	1	1	0	0	0	0	4
5281672	1	1	0	0	1	1	0	0	0	0	4
5280343	1	1	0	0	1	1	0	0	0	0	4

8742	1	0	0	1	1	1	0	0	0	0	4
5281618	1	0	0	1	1	1	0	0	0	0	4
5281222	1	0	0	1	1	1	0	0	0	0	4
5280863	1	1	0	0	1	1	0	0	0	0	4
5280804	1	0	1	0	1	1	0	0	0	0	4
5280459	1	0	1	0	1	1	0	0	0	0	4
12303220	1	0	0	0	1	1	0	1	0	0	4
5281643	1	0	1	0	1	1	0	0	0	0	4
157010024	1	0	0	1	1	1	0	0	0	0	4
8655	0	0	0	1	1	1	0	0	1	0	4
6047	1	0	0	0	1	1	0	0	1	0	4
6322	1	1	0	1	0	1	0	0	0	0	4
484757	1	0	0	0	0	1	0	1	0	0	3
637098	1	0	0	0	0	1	0	1	0	0	3
1060	0	0	0	1	1	1	0	0	0	0	3
5311498	1	0	1	0	0	1	0	0	0	0	3
5281766	1	0	0	0	1	1	0	0	0	0	3
5281728	1	0	0	0	0	1	0	1	0	0	3
1183	0	0	0	0	1	1	0	0	1	0	3
5793	1	0	0	0	1	1	0	0	0	0	3
5961	1	0	0	0	1	1	0	0	0	0	3
5280417	1	0	0	0	1	1	0	0	0	0	3
5276454	1	0	0	0	1	1	0	0	0	0	3
6036	1	0	0	0	1	1	0	0	0	0	3
11249520	1	0	0	0	1	1	0	0	0	0	3
689043	0	0	0	1	1	1	0	0	0	0	3
443651	1	0	0	0	1	1	0	0	0	0	3
656516	1	0	0	1	0	1	0	0	0	0	3
643733	0	0	0	0	1	1	0	1	0	0	3
637542	0	0	0	1	1	1	0	0	0	0	3
637540	0	0	0	1	1	1	0	0	0	0	3
6441280	1	0	0	0	1	1	0	0	0	0	3
11250133	1	0	0	0	0	1	0	1	0	0	3
441773	1	0	0	0	0	1	0	1	0	0	3
72193652	1	0	0	0	1	1	0	0	0	0	3
157009738	1	0	0	0	0	1	0	0	1	0	3
157009726	1	0	0	0	1	1	0	0	0	0	3
157009725	1	0	0	0	1	1	0	0	0	0	3
156614133	1	0	0	1	0	1	0	0	0	0	3
131752272	1	0	0	0	1	1	0	0	0	0	3
131752138	1	0	0	0	1	1	0	0	0	0	3
338	0	0	0	1	1	1	0	0	0	0	3
370	0	0	0	1	1	1	0	0	0	0	3
74977116	1	0	0	0	1	1	0	0	0	0	3
44256961	1	0	0	0	1	1	0	0	0	0	3
892	1	0	0	0	1	1	0	0	0	0	3
44256932	1	0	0	1	0	1	0	0	0	0	3
16131430	1	0	0	0	0	1	0	1	0	0	3
15714477	1	0	0	0	1	1	0	0	0	0	3
15385440	1	0	0	0	1	1	0	0	0	0	3
849	0	1	0	0	1	1	0	0	0	0	3
14311152	1	0	0	0	1	1	0	0	0	0	3

13990893	1	0	0	0	0	1	0	1	0	0	3
13831068	1	0	0	0	0	1	0	1	0	0	3
13751990	1	0	0	0	0	1	0	1	0	0	3
6054	0	0	1	0	1	1	0	0	0	0	3
19	0	0	0	1	1	1	0	0	0	0	3
6134	1	0	0	1	0	1	0	0	0	0	3
6306	0	1	0	0	1	1	0	0	0	0	3
119258	1	0	0	0	1	1	0	0	0	0	3
122738	1	0	0	0	0	1	0	1	0	0	3
107905	1	0	0	0	1	1	0	0	0	0	3
68245	1	0	0	0	1	0	0	1	0	0	3
64689	1	0	0	0	1	1	0	0	0	0	3
13250	0	0	0	0	1	1	0	0	1	0	3
147299	1	0	0	0	0	1	0	1	0	0	3
12025	0	0	0	0	1	1	0	1	0	0	3
157643	1	0	0	0	1	1	0	0	0	0	3
160521	1	0	0	0	1	1	0	0	0	0	3
169853	1	0	0	0	0	1	0	1	0	0	3
171548	1	0	0	0	0	1	0	0	1	0	3
10742	0	0	0	1	1	1	0	0	0	0	3
6255	1	0	0	1	0	1	0	0	0	0	3
439230	1	1	0	0	0	1	0	0	0	0	3
10393	1	0	0	0	1	1	0	0	0	0	3
237332	0	0	0	0	1	1	0	1	0	0	3
284	0	0	0	0	1	1	0	0	0	0	2
79803	0	0	0	0	1	1	0	0	0	0	2
8857	0	0	0	0	1	1	0	0	0	0	2
5284607	0	0	0	0	1	1	0	0	0	0	2
177	0	0	0	0	1	1	0	0	0	0	2
1054	1	0	0	0	0	1	0	0	0	0	2
6115	0	0	0	0	0	1	0	1	0	0	2
985	0	1	0	0	0	1	0	0	0	0	2
971	0	0	0	0	1	1	0	0	0	0	2
88000	1	0	0	0	0	1	0	0	0	0	2
938	0	0	0	0	1	1	0	0	0	0	2
8094	0	0	0	0	1	1	0	0	0	0	2
8892	0	0	0	0	1	1	0	0	0	0	2
8158	0	0	0	0	1	1	0	0	0	0	2
45109789	1	0	0	0	0	1	0	0	0	0	2
135413566	1	0	0	0	0	1	0	0	0	0	2
54682930	0	0	0	0	1	1	0	0	0	0	2
247	0	0	0	0	1	1	0	0	0	0	2
379	0	0	0	0	1	1	0	0	0	0	2
1130	0	0	0	0	0	1	0	1	0	0	2
264	0	0	0	0	1	1	0	0	0	0	2
10467	0	1	0	0	0	1	0	0	0	0	2
10690	1	0	0	0	0	1	0	0	0	0	2
5282767	0	1	0	0	0	1	0	0	0	0	2
5280795	0	0	0	0	1	1	0	0	0	0	2
5280845	0	0	0	0	1	1	0	0	0	0	2
5281127	0	1	0	0	0	1	0	0	0	0	2
493570	1	0	0	0	0	1	0	0	0	0	2

6202	1	0	0	0	0	0	0	1	0	0	2
5997	0	0	0	0	1	1	0	0	0	0	2
152059	0	0	0	0	1	1	0	0	0	0	2
5991	0	0	0	0	1	1	0	0	0	0	2
5280450	0	1	0	0	0	1	0	0	0	0	2
445639	0	1	0	0	0	1	0	0	0	0	2
5870	0	0	0	0	1	1	0	0	0	0	2
439195	0	0	0	0	1	1	0	0	0	0	2
445354	0	0	0	0	1	1	0	0	0	0	2
127	0	0	0	0	1	1	0	0	0	0	2
5780	1	0	0	0	0	1	0	0	0	0	2
637517	0	1	0	0	0	1	0	0	0	0	2
2969	0	0	0	0	1	1	0	0	0	0	2
3893	0	0	0	0	1	1	0	0	0	0	2
5570	0	0	0	0	1	1	0	0	0	0	2
5281	0	1	0	0	0	1	0	0	0	0	2
6857447	0	0	0	0	0	1	0	0	0	0	1
92094	0	0	0	0	0	1	0	0	0	0	1
53477794	0	0	0	0	0	1	0	0	0	0	1
53477891	0	0	0	0	0	1	0	0	0	0	1
5281243	0	0	0	0	0	1	0	0	0	0	1
446284	0	0	0	0	0	1	0	0	0	0	1
452110	1	0	0	0	0	0	0	0	0	0	1
11503	0	0	0	0	0	1	0	0	0	0	1
702	0	0	0	0	1	0	0	0	0	0	1
11005	0	0	0	0	0	1	0	0	0	0	1
5287939	0	0	0	0	0	1	0	0	0	0	1
222284	0	0	0	0	1	0	0	0	0	0	1
5280489	0	0	0	0	0	1	0	0	0	0	1
1031	0	0	0	0	1	0	0	0	0	0	1
5280934	0	0	0	0	0	1	0	0	0	0	1
13849	0	0	0	0	0	1	0	0	0	0	1
6405	0	0	0	0	1	0	0	0	0	0	1
14985	0	0	0	0	0	1	0	0	0	0	1
31260	0	0	0	0	1	0	0	0	0	0	1
5281235	0	0	0	0	0	1	0	0	0	0	1
6341	0	0	0	0	1	0	0	0	0	0	1
5748352	0	0	0	0	0	1	0	0	0	0	1
445580	0	0	0	0	0	1	0	0	0	0	1
6419725	0	0	0	0	0	1	0	0	0	0	1
7504	0	0	0	0	0	1	0	0	0	0	1
6436907	0	0	0	0	0	1	0	0	0	0	1
6560	0	0	0	0	1	0	0	0	0	0	1
10372299	0	0	0	0	0	1	0	0	0	0	1
753	0	0	0	0	1	0	0	0	0	0	1
1146	0	0	0	0	0	0	0	0	0	0	0
273	0	0	0	0	0	0	0	0	0	0	0
305	0	0	0	0	0	0	0	0	0	0	0
75228679	0	0	0	0	0	0	0	0	0	0	0
674	0	0	0	0	0	0	0	0	0	0	0
8471	0	0	0	0	0	0	0	0	0	0	0
8083	0	0	0	0	0	0	0	0	0	0	0

8060	0	0	0	0	0	0	0	0	0	0	0
8021	0	0	0	0	0	0	0	0	0	0	0
1045	0	0	0	0	0	0	0	0	0	0	0

Supplementary table 5 The antibodies used in this study.

Antibody	Company/Origin	Catalog number	Application	Dilution
anti-sirt1	CST	9475	Western Blot	1:250
anti-AC-stat3	CST	2523	Western Blot	1:500
anti-stat3	Proteintech	60199-1-Ig	Western Blot	1:500
anti-PSD95	Abcam	ab238135	Immunofluorescence	1:100
anti-PSD95	Abcam	ab238135	Western Blot	1:2000
anti-Synaptophysin	Abcam	ab32127	Western Blot	1:5000
anti-NQO1	Abcam	AB80588	Western Blot	1:10000
anti-GST	Sigma-Aldrich	G7781-25UL	Western Blot	1:2000
anti-CREB	CST	9197	Western Blot	1:1000
anti-CREB	CST	9197	Chromatin immunoprecipitation	1:50
anti-P-CREB	CST	9198	Immunofluorescence	1:400
anti-P-CREB	CST	9198	Western Blot	1:1000
anti-NRF2	Santa Cruz Biotechnology	SC-365949	Western Blot	1:1000
anti-iba1	Abcam	ab283319	Immunofluorescence	1:100
CoraLite488-conjugated Goat Anti-Rabbit IgG(H+L)	Proteintech	SA00013-2	Immunofluorescence	1:500
CoraLite594 –conjugated Goat Anti-Rabbit IgG(H+L)	Proteintech	SA00013-4	Immunofluorescence	1:500
anti-GAPDH	Proteintech	10494-1-AP	Western Blot	1:5000
HRP-conjugated Affinipure Goat Anti-Mouse IgG(H+L)	Proteintech	SA00001-1	Western Blot	1:5000
HRP-conjugated Affinipure Goat Anti-Rabbit IgG(H+L)	Proteintech	SA00001-2	Western Blot	1:5000

Supplementary table 6 AD Patient's specific information.

**Patient**	**Age**	**Gender**	**BMI**	**CDR Score**
134033	68	woman	24.56	1
191838	85	woman	25.54	1
295714	87	man	18.37	1
295158	76	woman	15.24	1
289709	70	woman	22.51	1
276486	63	man	26.08	1
251731	82	woman	17.77	2
292789	78	man	23.03	2
295155	68	man	22.22	2
131924	67	woman	21.09	2
290754	59	man	21.01	3
289690	81	woman	20.89	3
295603	55	woman	26.10	3

Supplementary table 7 Specific primer sequences utilized for the qPCR analysis.

Gene symbol	Forward Primer	Reverse Primer
Gapdh	TGGCCTTCCGTGTTCCTAC	GAGTTGCTGTTGAAGTCGCA
Dlg4	TGAGATCAGTCATAGCAGCTACT	CTTCCTCCCCTAGCAGGTCC
syp	GAGAGAACAACAAAGGGCCAA	GCGGATGAGCTAACTAGCCAC
Il1r1	GTGCTACTGGGGCTCATTTGT	GGAGTAAGAGGACACTTGCGAAT
Il1beta	GCAACTGTTCCTGAACTCAACT	ATCTTTTGGGGTCCGTCAACT
Tnf	GACGTGGAACTGGCAGAAGAG	TTGGTGGTTTGTGAGTGTGAG
Nrf2	CTGAACTCCTGGACGGGACTA	CGGTGGGTCTCCGTAAATGG
Nqo1	TGGCCGAACACAAGAAGCTG	GCTACGAGCACTCTCTCAAACC
Mgst1	CTCAGGCAGCTCATGGACAAT	GTTATCCTCTGGAATGCGGTC
Sirt1	GCTGACGACTTCGACGACG	TCGGTCAACAGGAGGTTGTCT
Ubqln	GGACTTACGGTTCACCTTGTC	CTTCCAGGGGCATTTGTTTGC
Axl	GGAACCCAGGGAATATCACAGG	AGTTCTAGGATCTGTCCATCTCG
Fer	GTGTGATGTGATCTCTGTGGG	GCAGTTCCTGTGCCTCTATCC
Sp1	AGGGTCCGAGTCAGTCAGG	CTCGCTGCCATTGGTACTGTT
Park7	AGCCGGGATCAAAGTCACTG	GGTCCCTGCGTTTTTGCATC
Cnr1	AAGTCGATCTTAGACGGCCTT	TCCTAATTTGGATGCCATGTCTC
Cx3cr1	TCTGGACTAATCCTGAGGGTG	GCCTGTTGGTTATTGGAACTCTC
